# Hepcidin inhibits Smad3 phosphorylation in hepatic stellate cells by impeding ferroportin-mediated regulation of Akt

**DOI:** 10.1038/ncomms13817

**Published:** 2016-12-22

**Authors:** Chang Yeob Han, Ja Hyun Koo, Sung Hoon Kim, Sara Gardenghi, Stefano Rivella, Pavel Strnad, Se Jin Hwang, Sang Geon Kim

**Affiliations:** 1 College of Pharmacy and Research Institute of Pharmaceutical Sciences, Seoul National University, Seoul 08826, Korea; 2 College of Medicine, Hanyang University, Seoul 04763, Korea; 3 Department of Pediatrics, Division of Hematology-Oncology, Weill Cornell Medical College, New York, New York 10021, USA; 4 IZKF and Department of Internal Medicine III, University Hospital Aachen, Aachen 52074, Germany

## Abstract

Hepatic stellate cell (HSC) activation on liver injury facilitates fibrosis. Hepatokines affecting HSCs are largely unknown. Here we show that hepcidin inhibits HSC activation and ameliorates liver fibrosis. We observe that hepcidin levels are inversely correlated with exacerbation of fibrosis in patients, and also confirm the relationship in animal models. Adenoviral delivery of hepcidin to mice attenuates liver fibrosis induced by CCl_4_ treatment or bile duct ligation. In cell-based assays, either hepcidin from hepatocytes or exogenous hepcidin suppresses HSC activation by inhibiting TGFβ1-mediated Smad3 phosphorylation via Akt. In activated HSCs, ferroportin is upregulated, which can be prevented by hepcidin treatment. Similarly, ferroportin knockdown in HSCs prohibits TGFβ1-inducible Smad3 phosphorylation and increases Akt phosphorylation, whereas ferroportin over-expression has the opposite effect. HSC-specific ferroportin deletion also ameliorates liver fibrosis. In summary, hepcidin suppresses liver fibrosis by impeding TGFβ1-induced Smad3 phosphorylation in HSCs, which depends on Akt activated by a deficiency of ferroportin.

## Abstract (web summary)

The peptide hormone hepcidin is released from hepatocytes and regulates iron homoeostasis. Here, the authors show that hepcidin also regulates the activation of hepatic stellate cells (HSCs) in mouse models of liver fibrosis by reducing ferroportin expression and inhibiting the HSC response to TGFβ.

Emerging evidence suggests the importance of crosstalk between neighbouring cells and hepatic stellate cells (HSCs) in liver biology^1^,^2^,^3^,^4^. The microenvironments in the space of Disse consisting of parenchymal cells and sinusoidal endothelial cells contribute to the maintenance of the characteristics of quiescent HSCs in normal rat liver^2^, implying that mediators derived from hepatocytes play a role in preserving HSCs in a quiescent state. In disease conditions, HSCs undergo transdifferentiation from quiescent cells to myofibroblast-like cells, and the activated cells are then the primary source of extracellular matrix (ECM) proteins on liver injury and mainly contribute to liver fibrosis^5^,^6^. Hence, altered paracrine activities of hepatocytes and the subsequent derangement of cell–cell communication may be crucial in the initiation and perpetuation of HSC activation in the progression of liver disease. Despite the crosstalk between hepatocytes and HSCs, hepatokines affecting the neighbouring HSCs are largely unknown.

Liver fibrosis due to chronic viral hepatitis, hepatotoxicants and alcoholic or non-alcoholic fatty liver disease may proceed to cirrhosis, which is one of the major causes of morbidity and mortality worldwide. The deposition of iron and the consequent hemosiderosis are common features of liver fibrosis, implying that iron overload may be a major risk factor for liver disease progression^7^. Moreover, iron accumulation may expedite tissue injury by promoting oxidative stress^7^. Hepcidin (*HAMP*), a polypeptide hormone mainly produced by hepatocytes, regulates iron homoeostasis. Since hepcidin expression is tightly controlled physiologically, iron loading triggers hepcidin to maintain systemic iron homoeostasis, which is accomplished by inhibiting intestinal iron absorption and iron recycling^8^. In this event, hepcidin interacts with an iron exporter, ferroportin (FPN, *SLC40A1*), facilitating its degradation^9^. Despite the information on the presence of FPN in rat HSCs^10^, nothing is known on the paracrine activity of hepcidin on HSCs.

A limitation of the current anti-fibrotic therapy is that medicinal candidates cannot specifically target HSCs and may frequently be toxic to parenchymal cells^11^. The strategy to repair the endogenous regulatory system between hepatocytes and HSCs may provide the optimal method for therapy with minimal adverse effects.

Here we report that the hepcidin produced from hepatocytes controls the biology of neighbouring HSCs, serving as an anti-fibrotic hepatokine. Examination of hepcidin levels in patients with liver fibrosis showed the clinical impact of hepcidin repression on exacerbation of fibrosis. Diverse animal models and cell-based assays were used to verify the paracrine action of hepcidin on HSCs and elucidate the underlying molecular basis. Our finding that hepcidin inhibits transforming growth factor β1 (TGFβ1)-mediated Smad3 phosphorylation by degrading FPN that is upregulated and responsible for the suppression of Akt signalling in activated HSCs, offers the novel therapeutic approaches to overcome liver fibrosis.

## Results

### Inverse correlation between hepcidin and liver fibrosis

**Figure 1:**
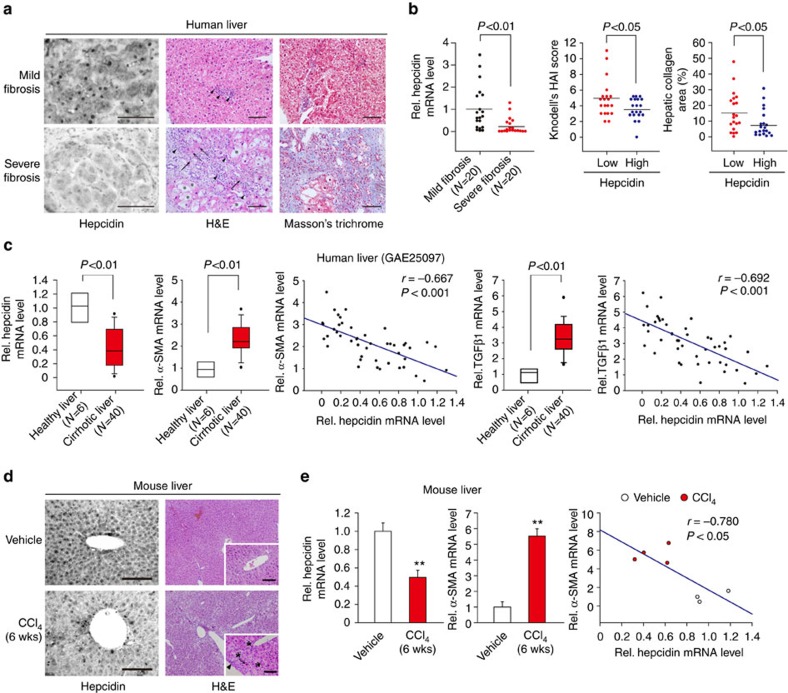
Inverse correlation between hepcidin expression and the severity of liver fibrosis. (**a**,**b**) Immunohistochemistry (IHC) and qRT–PCR assays for hepcidin in fibrosis patients. IHC for human hepcidin, hematoxylin–eosin (H&E) and Masson's trichrome stainings on the liver sections from representative patients with mild or severe fibrosis. Asterisks indicate ballooning degeneration of hepatocytes, whereas arrowheads do inflammatory cell infiltration. Thin arrows represent eosinophilic necrosis of hepatocyte, whereas arrows do fibrosis in portal area (scale bar, 100 μm). Ishak fibrosis scores, Knodell's histological activity index and collagen area had been previously determined for the samples. Statistical significance of the differences between mild and severe fibrosis (or hepcidin median low and high groups) was analysed using unpaired two-sample Student's *t*-test (*N*=20 each). (**c**) Transcript levels of hepcidin, α-SMA and TGFβ1 in healthy individuals or cirrhosis patients (GSE25097), and the inverse correlation of hepcidin mRNA with either α-SMA or TGFβ1 mRNA. Data were shown as box and whisker plot. Box, interquartile range; whiskers, 5–95 percentiles; horizontal line within box, median. Statistical significance of the differences between healthy individuals and cirrhosis patients was determined by unpaired two-sample Student's *t*-test (*N*=6 or 40 each). (**d**) IHC for hepcidin using anti-mouse hepcidin antibody and H&E staining on the liver sections from representative mice treated with vehicle or CCl_4_ (0.6 ml kg^−1^ body weight, i.p., twice a week, for 6 weeks). Asterisks indicate widespread swelling of hepatocytes, whereas an arrow does hepatic necrosis and an arrowhead represents inflammatory cell infiltration (scale bar, 100 μm). (**e**) Hepcidin and α-SMA transcript levels in the mice treated as above. Data represent the mean±s.e.m. (*N*=3 or 4). Statistical significance of the differences between each treatment and vehicle group (**P<0.01) was determined by unpaired two-sample Student's *t*-test.

To identify the biological relevance of hepcidin in liver fibrosis during clinical situations, the expression of hepcidin in the liver was compared between patients with mild or severe fibrosis. Immunohistochemical analyses revealed that hepcidin expression was lower in patients with severe fibrosis than mild fibrosis (Fig. 1a). Moreover, hepcidin transcript levels also decreased, as the disease progressed in severity (Fig. 1b). When we compared the degrees of fibrosis in the groups of median low or median high hepcidin, hepcidin expression was reciprocal to the means of either modified Knodell's histological activity index or hepatic collagen area.

In the analysis of a human GEO database (GEO accession code GSE25097) available in public domain, hepcidin messenger RNA (mRNA) levels were also lower in cirrhotic livers than in healthy livers. As expected, the mRNA levels of α-smooth muscle actin (α-SMA, a well-established marker of HSC activation) and TGFβ1 (a representative profibrogenic cytokine) were both elevated in cirrhotic liver and inversely correlated with the hepcidin expression (Fig. 1c). These indicate that hepcidin is downregulated in the liver of fibrosis patients as the disease worsens, raising the hypothesis that the loss of hepcidin plays a role in the activation of HSCs.

Next, hepcidin expression was monitored in different animal models of toxicant-induced liver fibrosis. In mice treated with carbon tetrachloride (CCl_4_) for 6 weeks, hepcidin protein and mRNA levels were both decreased in the liver (Fig. 1d,e). In addition, hepcidin transcript levels inversely correlated with those of α-SMA (Fig. 1e). Repression of hepcidin expression was also observed in the livers of rats treated with dimethylnitrosamine for 4 weeks (Supplementary Fig. 1). Immunostainings using hepcidin knockout (KO) and knockdown samples verified specificities of the antibodies (Supplementary Fig. 2). The data indicate that the expression of hepcidin is downregulated in fibrotic liver likely in association with HSC activation.

### Inhibition of liver fibrosis by hepcidin over-expression

**Figure 2:**
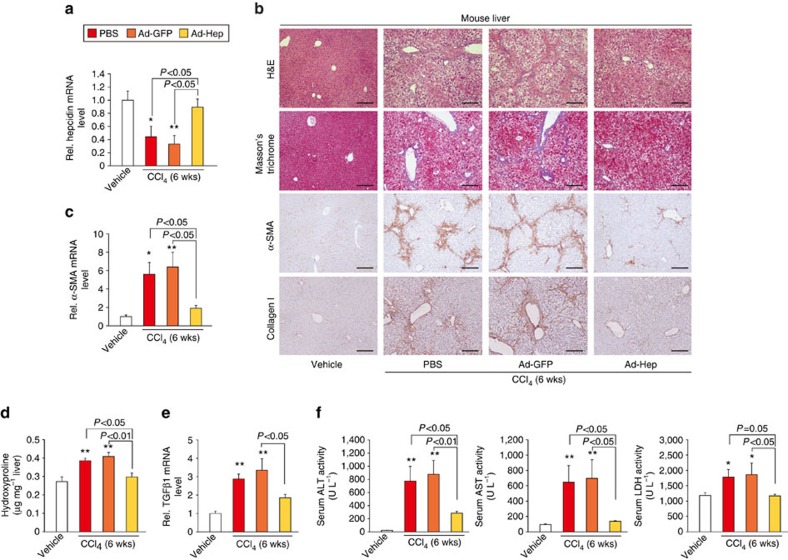
Inhibition of CCl_4_-induced liver fibrosis by hepcidin. (**a**) Hepatic hepcidin transcript levels in mice treated with vehicle or CCl_4_ (0.6 ml kg^−1^ body weight, i.p., twice a week, for 6 weeks) in combination with tail vein injection of PBS, Ad-GFP or Ad-Hep (*N*=5 or 6). Details for treatment schedule are provided in the Methods section. (**b**) H&E, Masson's trichrome stainings and IHC for α-SMA or collagen type 1 were done on the liver tissues of mice treated as above (scale bar, 100 μm). Representative images derived from replicate experiments (*N*=3 each) were shown. (**c**) qRT–PCR assays for α-SMA in the liver of mice as in **a**. (**d**) Collagen content was measured by biochemical determination of hydroxyproline (per mg of liver) in the liver of mice as in **a**. (**e**) qRT–PCR assays for TGFβ1 in the liver of mice as in **a**. (**f**) Liver function was assessed by ALT, AST and LDH activities in serum of mice as in **a**. For **a** and **c**–**f**, data represent the mean±s.e.m. Statistical significance of the differences between each treatment group and vehicle (*P<0.05, **P<0.01) was determined by analysis of variance (Bonferroni's or LSD method).

On the basis of the decrease in hepcidin expression in fibrotic liver, we next examined whether the forced expression of hepcidin inhibits liver fibrosis in animal models. Adenoviral hepcidin (Ad-Hep) delivery system was utilized to mice repetitively exposed to CCl_4_ for 6 weeks. Quantitative reverse transcription PCR (qRT–PCR) assays ascertained hepcidin over-expression in the liver (Fig. 2a). Hematoxylin and eosin (H&E) and Masson's trichrome staining assays revealed that hepcidin over-expression inhibited CCl_4_-induced liver fibrosis as compared with controls (Fig. 2b). Consistently, enforced expression of hepcidin notably attenuated increases in the levels of α-SMA and collagen type 1 (Col-1A1), the most abundant ECM protein in the fibrotic liver tissue (Fig. 2b). Similarly, α-SMA mRNA levels were decreased (Fig. 2c). Consistently, Ad-Hep treatment prevented the increase in hydroxyproline content (Fig. 2d). Hepatic TGFβ1 mRNA levels were also mitigated (Fig. 2e). In blood biochemistry analyses, alanine aminotransferase (ALT), aspartate aminotransferase (AST) and lactate dehydrogenase (LDH) activities were all significantly diminished by hepcidin over-expression, indicative of improvement of liver function (Fig. 2f).

**Figure 3:**
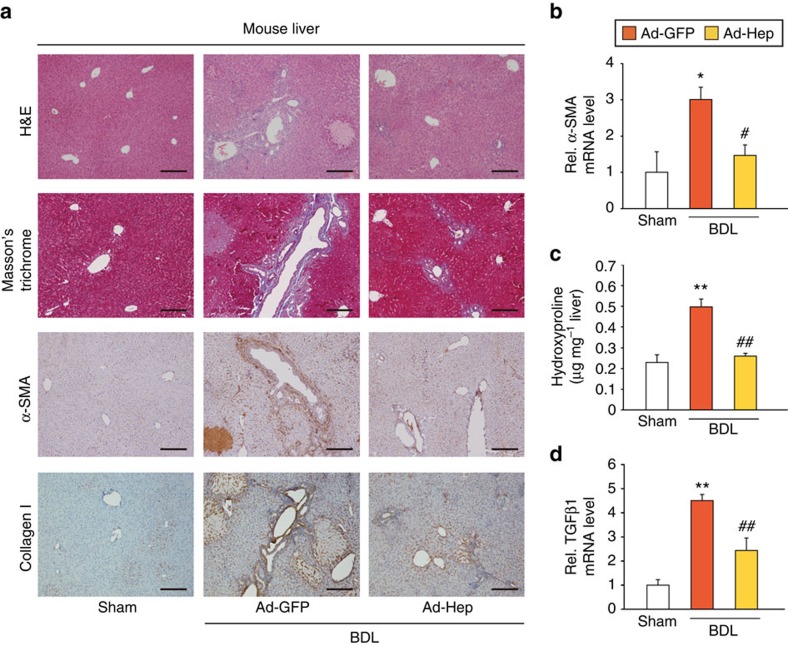
Inhibition of BDL-induced liver fibrosis by hepcidin. (**a**) H&E, Masson's trichrome stainings and IHC for α-SMA or collagen type 1 were done on the liver tissues of mice operated with sham or BDL (for 2 weeks) in combination with tail vein injection of Ad-GFP or Ad-Hep (*N*=5 or 6; scale bar, 100 μm). Details for treatment schedule are provided in the Methods section. Representative images derived from replicate experiments (*N*=3 each) were shown. (**b**) qRT–PCR assays for α-SMA in the liver of mice as in **a**. (**c**) Determination of hydroxyproline content in the liver of mice as in **a**. (**d**) qRT–PCR assays for TGFβ1 in the liver of mice as in **a**. For **b**–**d**, data represent the mean±s.e.m. (*N*=5 or 6). Statistical significance of the differences between each treatment group and sham (*P<0.05, **P<0.01), or BDL+Ad-GFP (^#^*P*<0.05, ^##^*P*<0.01) was determined by analysis of variance (Bonferroni's method).

To further analyse the anti-fibrotic effect of hepcidin, we employed the bile duct ligation (BDL) mouse model of liver fibrosis, another well-established model. Adenoviral delivery of hepcidin prohibited the development of liver fibrosis in BDL-operated mice, as evidenced by histopathological improvement and the changes in Masson's trichrome (Fig. 3a). Repression of α-SMA gene expression was verified by the results of immunohistochemistry and qRT–PCR assays (Fig. 3a,b). Similarly, hepatic collagen-staining intensities and hydroxyproline content were lessened by Ad-Hep delivery (Fig. 3a,c). TGFβ1 mRNA levels were also suppressed (Fig. 3d).

To validate the biological impact of Ad-Hep delivery, we additionally used an animal model of toxicant-induced hepatitis. A single injection of CCl_4_ to mice decreased hepcidin expression in the liver with a reciprocal increase in TGFβ1 mRNA at 24 h (Supplementary Fig. 3a,b). An injection of Ad-Hep to mice before CCl_4_ treatment ameliorated liver histopathology (that is, reduction of hepatocyte necrosis and inflammatory cell infiltration, and maintenance of sinusoidal structure) and significantly inhibited the increases in α-SMA or TGFβ1 expression (Supplementary Fig. 3c–e). Consistently, hepcidin over-expression improved blood biochemical parameters (Supplementary Fig. 3f), suggesting that hepcidin also has an inhibitory effect on the early pathologic event leading to the liver fibrogenesis. qRT–PCR assays for α-SMA confirmed that Ad-Hep infection inhibited fibrogenic gene induction in a dose-dependent manner (Supplementary Fig. 3g). All of these results demonstrate that hepcidin has the capability to inhibit the liver fibrosis development and progression.

### Inhibition of HSCs by hepcidin from hepatocytes

**Figure 4:**
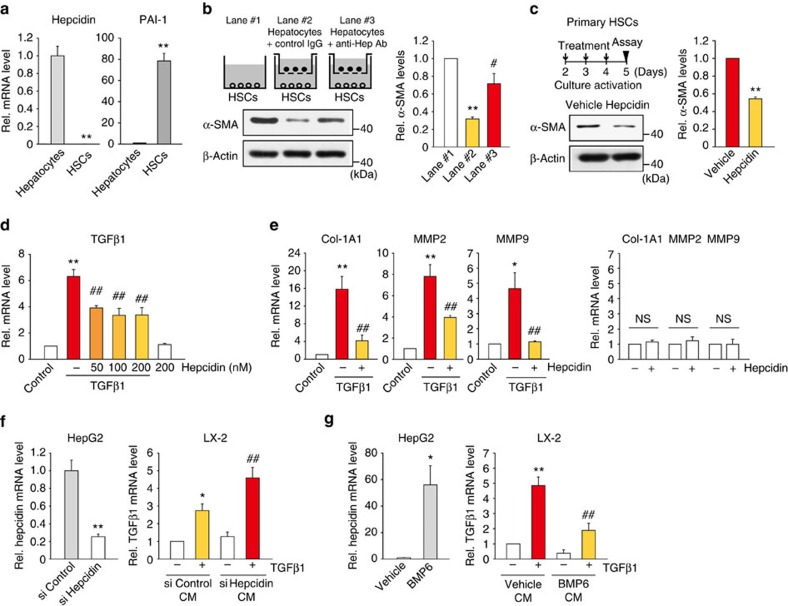
Inhibition of HSC activation and TGFβ1 effect by hepcidin. (**a**) Hepcidin and plasminogen activator inhibitor 1 (PAI-1) mRNA levels in rat primary hepatocytes or rat primary HSCs. (**b**) Immunoblotting for α-SMA. HSCs freshly isolated from the rat were maintained in monoculture (lane 1), co-culture with hepatocytes in the presence of control IgG (lane 2) or anti-hepcidin polyclonal antibody (2 μg ml^−1^; lane 3) for 5 days. Data represent the mean±s.e.m. of three separate experiments. Statistical significance of the differences between each groups and HSCs alone (**P<0.01), or HSCs+Hepatocyte co-culture+Control IgG (^#^*P*<0.05) was determined by analysis of variance (ANOVA; Bonferroni's method). (**c**) Immunoblotting for α-SMA. Rat primary HSCs were cultured for 2 days, and then were daily treated with vehicle or 100 nM recombinant murine hepcidin for 3 days. Data represent the mean±s.e.m. of three separate experiments. Statistical significance of the differences between each treatment and vehicle group (**P<0.01) was determined by unpaired two-sample Student's *t*-test. (**d**) qRT–PCR assays for TGFβ1. LX-2 cells were exposed to recombinant human hepcidin for 3 h, and then continuously treated with 5 ng ml^−1^ TGFβ1 for additional 12 h. (**e**) qRT–PCR assays for Col-1A1, matrix metalloproteinase (MMP)-2, and MMP9. The cells were similarly treated as in **d** (hepcidin, 100 nM). (**f**) The effect of conditioned media (CM) collected from HepG2 cells deficient of hepcidin (siRNA). qRT–PCR assays were done on HepG2 cells transfected with control siRNA or hepcidin siRNA for 48 h (left) or LX-2 cells incubated with the respective medium collected from the HepG2 cells with or without TGFβ1 for 12 h (right). (**g**) qRT–PCR assays. The cells were similarly treated as in **f** except for 20 μM BMP6 treatment for 12 h (left). For **a** and **d**–**g**, data represent the mean±s.e.m. of at least three separate experiments. Statistical significance of the differences between hepatocytes and HSCs (**P<0.01; unpaired two-sample Student's *t*-test), or between each treatment and control group (*P<0.05, **P<0.01) or TGFβ1 (^##^*P*<0.01) was determined by ANOVA (Bonferroni's or LSD method).

To address hepcidin-mediated communications between hepatocytes and HSCs, we examined whether hepcidin produced by healthy hepatocytes can prevent the HSC activation *in vitro*. Hepcidin was expressed mainly in rat primary hepatocytes, but not in activated HSCs (Fig. 4a), supporting its paracrine origin. Plasminogen activator inhibitor 1 expression verified the activation of HSCs. Co-culture with hepatocytes prevented HSCs from inducing α-SMA expression and this inhibition was ablated by the addition of an anti-hepcidin antibody (Fig. 4b). Moreover, hepcidin treatment inhibited α-SMA expression in HSCs during the culture activation (Fig. 4c). Treatment of HSCs with hepcidin did not induce apoptosis or cell proliferation (Supplementary Fig. 4a,b), allowing the focus on fibrogenesis of HSCs.

TGFβ1, the key profibrogenic cytokine, plays a vital role in the HSC activation and liver fibrogenesis^12^. In the present study, LX-2, a human HSC line, was used for the mechanistic experiments (this cell line has key features of HSCs despite limitations due to immortalization^13^), and the main findings were confirmed using rat or mouse primary HSCs. We first tested whether hepcidin inhibits TGFβ1 signalling pathway in HSCs. Hepcidin treatment (at physiologically relevant concentrations)^14^ significantly lessened not only the level of TGFβ1 mRNA in LX-2 cells, but those of TGFβ1-inducible target gene transcripts (Col-1A1, matrix metalloproteinase (MMP)-2, and MMP9; Fig. 4d,e). Hepcidin alone had no effect on the basal gene expression. In subsequent experiments, we used TGFβ1 mRNA as an assay indicator. TGFβ1 mRNA levels were further enhanced after the culture of LX-2 cells with a conditioned medium (CM) collected from HepG2 cells deficient of hepcidin as compared with control CM (Fig. 4f). Consistently, the expression of TGFβ1 was reduced in the cells treated with CM from HepG2 cells that had been exposed to bone morphogenetic protein 6 (a hepcidin inducer; Fig. 4g), strengthening the paracrine effect of hepcidin on HSCs. Our results raised the possibility that hepcidin produced from hepatocytes inhibits the activation of HSCs, which may be mediated by inhibition of the TGFβ1 signalling pathway.

### Inhibition of TGFβ1-Smad3 pathway by hepcidin

**Figure 5:**
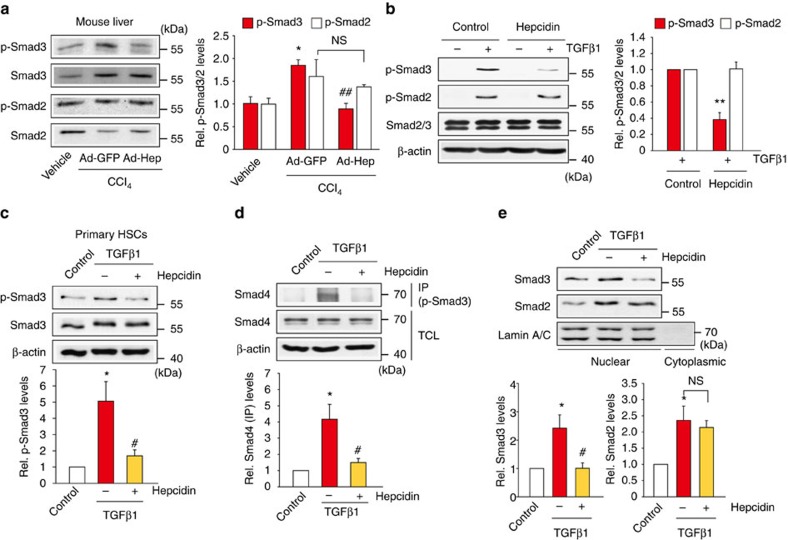
Inhibition of Smad3 phosphorylation in HSCs by hepcidin. (**a**) Immunoblottings for p-Smad2/3 in the liver. Mice were treated with vehicle or CCl_4_ (0.6 ml kg^−1^ body weight, i.p., 24 h) 6 days after a tail vein injection of Ad-GFP or Ad-Hep. Details for treatment schedule are provided in the Methods section. Relative protein levels were assessed by scanning densitometry of the immunoblots. Data represent the mean±s.e.m. of at least three animals. Statistical significance of the differences between each treatment group and vehicle (*P<0.05), or CCl_4_+Ad-GFP (^##^*P*<0.01) was determined by analysis of variance (ANOVA ; Bonferroni's method; NS, not significant). (**b**) Immunoblottings for p-Smad2/3. LX-2 cells were exposed to 100 nM hepcidin for 3 h and continuously treated with 5 ng ml^−1^ TGFβ1 for 20 min. Data represent the mean±s.e.m. of three separate experiments. Statistical significance of the differences between each treatment and TGFβ1 group (**P<0.01) was determined by unpaired two-sample Student's *t*-test. (**c**) Immunoblottings for p-Smad3. Rat primary HSCs were treated as described in **b**. (**d**) Immunoprecipitation and immunoblotting assay. Smad4 was immunoblotted on p-Smad3 immunoprecipitates of LX-2 cells treated as in **b** (TGFβ1 treatment for 1 h). (**e**) Immunoblottings for nuclear Smad2/3. Nuclear and cytoplasmic fractions were prepared from LX-2 cells treated as in **d**. Immunoblotting for Lamin A/C confirms the purity of nuclear fractions and equal protein loading. For **c**–**e**, data represent the mean±s.e.m. of three separate experiments. Statistical significance of the differences between each treatment and control group (*P<0.05) or TGFβ1 (^#^*P*<0.05) was determined by ANOVA (Bonferroni's method; NS, not significant).

Ligand activation of TGFβ1 receptor facilitates the phosphorylation of Smad3 and Smad2 in a canonical pathway^15^. As an effort to find hepcidin target and the associated signalling pathway, we monitored the status of Smad3 or Smad2 phosphorylation in the liver tissue or HSCs. In mice exposed to CCl_4_, Ad-Hep infection notably inhibited Smad3 phosphorylation with minimal inhibition of Smad2 phosphorylation (Fig. 5a). Consistently, treatment of LX-2 cells with hepcidin predominantly ablated TGFβ1-inducible Smad3 phosphorylation (Fig. 5b). The ability of hepcidin to specifically inhibit Smad3 was also confirmed in rat primary HSCs (Fig. 5c). Consistently, hepcidin treatment abolished Smad3 and Smad4 binding elicited by TGFβ1, an event occurring before their nuclear translocation (Fig. 5d). The predominant inhibition of Smad3 by hepcidin was verified by the absence of Smad3, but not Smad2, in the nuclear fractions (Fig. 5e). Our results demonstrate that hepcidin inhibits Smad3 phosphorylation downstream from the TGFβ1 receptor activation in HSCs, which may account for the anti-fibrotic effect of hepcidin.

### Akt-mediated inhibition of Smad3 phosphorylation by hepcidin

**Figure 6:**
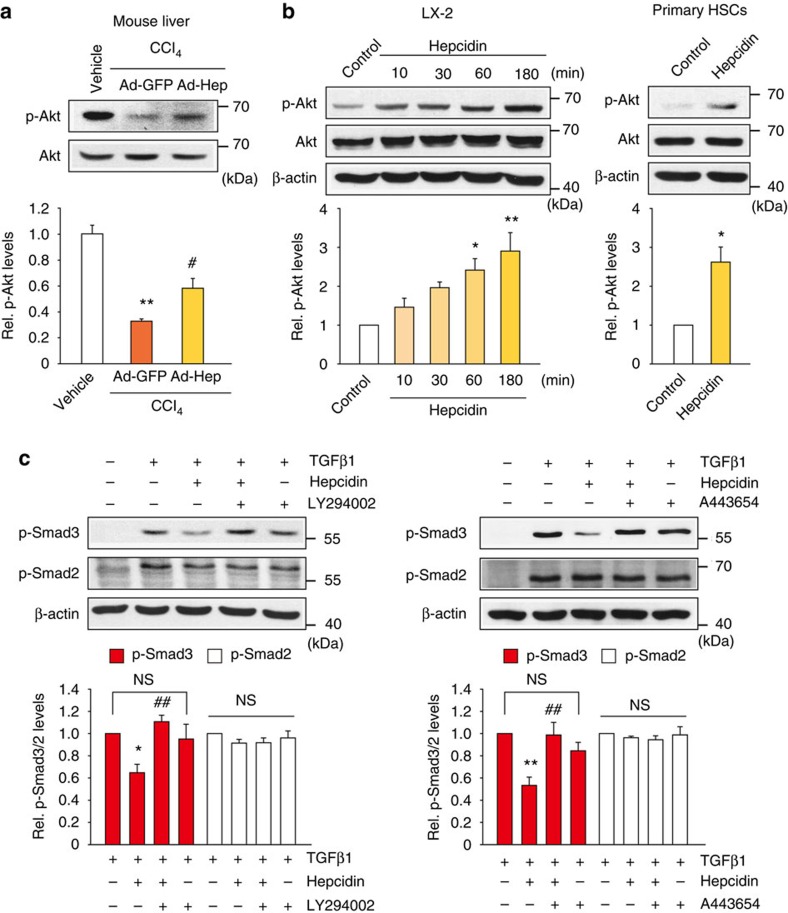
The effect of hepcidin on Akt and its role in Smad3 inhibition. (**a**) Immunoblotting for p-Akt. Akt phosphorylated at Thr308 and total Akt were immunoblotted on the liver homogenates of mice treated as described in Fig. 5a. Data represent the mean±s.e.m. of at least three animals. Statistical significance of the differences between each treatment group and vehicle (**P<0.01), or CCl_4_+Ad-GFP (^#^*P*<0.05) was determined by analysis of variance (ANOVA; LSD method). (**b**) Immunoblotting for p-Akt. LX-2 cells were treated with 100 nM hepcidin for the indicated times (left) or in rat primary HSCs incubated with hepcidin for 1 h (right). (**c**) Immunoblottings for p-Smad3 and p-Smad2. The cells were treated with hepcidin for 3 h and continuously incubated with 10 μM LY294002 or 1 μM A443654 for 10 min, followed by TGFβ1 treatment for 20 min. For **b** and **c**, data represent the mean±s.e.m. of three separate experiments. Statistical significance of the differences between each treatment groups (*P<0.05, **P<0.01) or TGFβ1+hepcidin (^##^*P*<0.01) was determined by ANOVA (Bonferroni's or LSD method; NS, not significant).

The TGFβ receptor signalling may crosstalk with other signals for the activation of Smad3 and Smad2. Since Akt may modulate Smads^16^,^17^, we hypothesized that hepcidin affects the activity of Akt in HSCs. In the liver of mice, Ad-Hep infection significantly prevented the decrease in p-Akt level caused by CCl_4_ treatment (Fig. 6a). Consistently, hepcidin treatment increased p-Akt in LX-2 cells in a time-dependent manner (Fig. 6b, left). A similar increasing effect was observed in rat primary HSCs (Fig. 6b, right). We then determined whether an increase in p-Akt by hepcidin was responsible for the inhibition of Smad3 phosphorylation in HSCs. Treatment with either LY294002 (a phosphatidyl inositol 3-kinase (PI3K) inhibitor) or A443654 (a pan-Akt inhibitor) resulted in a rescue of TGFβ1-inducible Smad3 phosphorylation (Fig. 6c). We additionally examined the knockdown effects of Akt isoforms. Of three Akt isoforms (Akt1, Akt2 and Akt3), Akt3 is predominantly expressed in the brain^18^. So, we focused on Akt1 and Akt2, and found that either siAkt1 or siAkt2 abolished hepcidin effect on p-Smad3 (Supplementary Fig. 5a), supporting that both isoforms may be required for the effect of hepcidin. All of these results provide strong evidence that hepcidin inhibition of Smad3 phosphorylation in HSCs may depend on the increase in the Akt activity.

### Inhibition of FPN and TGFβ1 pathway in HSCs by hepcidin

**Figure 7:**
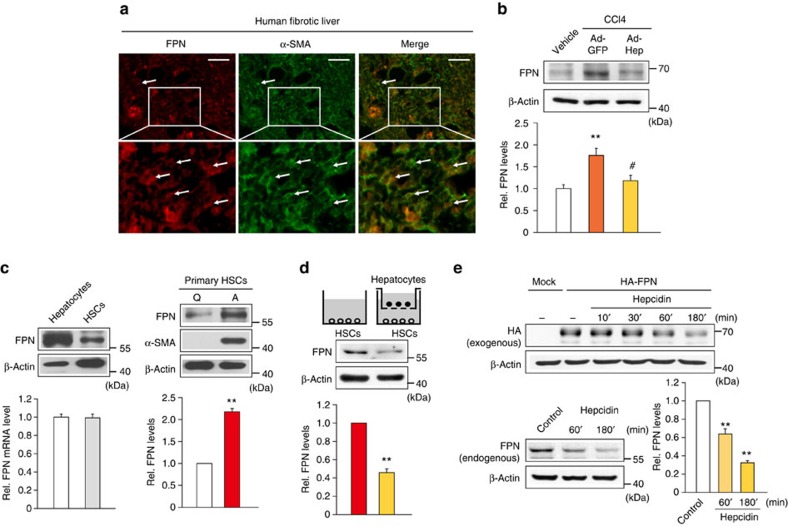
Hepcidin regulation of FPN in HSCs. (**a**) Double immunofluorescence staining of FPN and α-SMA in a fibrotic liver section from patients as described in Fig. 1a. The proteins were stained with Cy3- and Alexa488-conjugated secondary antibodies, respectively. Arrows indicate co-localization of FPN and α-SMA in morphologically activated HSCs (scale bar, 50 μm). (**b**) FPN expression in the liver of mice treated as described in Fig. 5a. Data represent the mean±s.e.m. of at least three animals. Statistical significance of the differences between each treatment group and vehicle (**P<0.01), or CCl_4_+Ad-GFP (^#^*P*<0.05) was determined by analysis of variance (ANOVA; LSD method). (**c**) FPN expression in rat primary hepatocytes and rat primary HSCs (left), or in quiescent (freshly isolated, day 0) and activated (day 6 after culture) HSCs (right). (**d**) FPN expression in rat primary HSCs cultured alone or co-cultured with hepatocytes for 5 days. For **c** and **d**, data represent the mean±s.e.m. of three separate experiments. Statistical significance of the differences between quiescent (Q) and activated (A) HSCs in **c** (**P<0.01), and between HSCs alone and co-culture with hepatocytes in **d** (**P<0.01) was determined by unpaired two-sample Student's *t*-test. (**e**) Immunoblottings for HA-tagged or endogenous FPN. FPN was measured on the lysates of LX-2 cells transfected with Mock or HA-FPN, and treated with 100 nM hepcidin (upper), or of those treated with hepcidin for the indicated times (lower). Data represent the mean±s.e.m. of three separate experiments. Statistical significance of the differences between each treatment and control group (**P<0.01) was determined by ANOVA (Bonferroni's method).

Hepcidin exerts its function for iron homoeostasis by binding to its receptor FPN^9^. Considering the relation of hepcidin with FPN, and the lack of information about the effect of hepcidin on FPN in HSCs, we wondered the role of FPN decrease in the inhibition of HSCs by hepcidin. In fibrosis patients, there was the simultaneous staining of FPN and α-SMA in liver sections (Fig. 7a). FPN transcript levels were significantly enhanced in the liver of cirrhosis patients compared with healthy individuals (GEO accession code GSE25097; Supplementary Fig. 6), which may reflect the adaptive receptor upregulation after a decrease in ligand level. The increase of FPN mRNA may also be associated with inflammatory condition^19^. A positive correlation existed between FPN and TGFβ1 levels. Consistently, the expression of FPN was enhanced by CCl_4_ treatment in the liver of mice, which was significantly inhibited by Ad-Hep infection (Fig. 7b). Immunoblottings and qRT–PCR assays revealed FPN expression in HSCs, as well as hepatocytes (Fig. 7c, left). Moreover, FPN levels were augmented in culture-activated HSCs (day 6) compared with quiescent HSCs (day 0; Fig. 7c, right). Hepatocyte co-culture reduced FPN expression in HSCs (Fig. 7d). Similarly, treatment of LX-2 cells with hepcidin facilitated the degradation of endogenous FPN, as well as ectopically expressed HA-tagged FPN (Fig. 7e).

**Figure 8:**
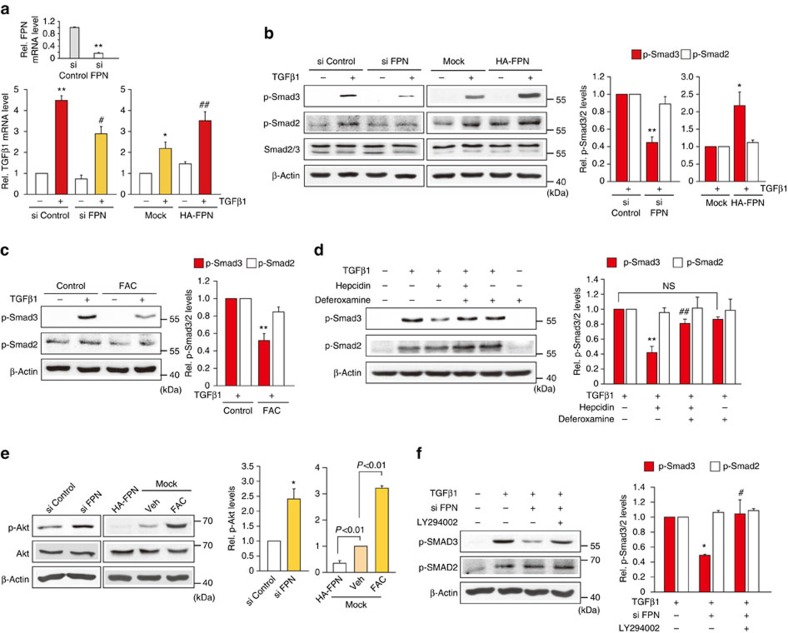
The effects of FPN modulations on TGFβ1 signalling pathway. (**a**) qRT–PCR assays for TGFβ1 after FPN modulations. LX-2 cells were transfected with control siRNA or FPN siRNA for 48 h (left), or with Mock or HA-FPN for 24 h (right), followed by TGFβ1 treatment for 12 h. Inset shows FPN knockdown. Data represent the mean±s.e.m. of at least three separate experiments. Statistical significance of the differences between each treatment and control group (*P<0.05, **P<0.01) or TGFβ1 (^#^*P*<0.05, ^##^*P*<0.01) was determined by analysis of variance (ANOVA; Bonferroni's or LSD method). (**b**) Immunoblottings for p-Smad2 and p-Smad3 in LX-2 cells transfected as in **a** (TGFβ1 treatment for 20 min). (**c**,**d**) Immunoblottings for p-Smad2 and p-Smad3. For **c**, LX-2 cells were exposed to ferric ammonium citrate (FAC, 10 μM) for 30 min and continuously treated with 5 ng ml^−1^ TGFβ1 for 20 min. For **d**, the cells were exposed to hepcidin for 3 h after deferoxamine treatment (100 μM for 3 h), and were treated with TGFβ1 as above. (**e**) Immunoblottings for p-Akt in LX-2 cells transfected as in **a** (left) or in the cells transfected with HA-FPN or Mock for 24 h. Mock-transfected cells were treated with vehicle or FAC for 30 min (right). Data represent the mean±s.e.m. of three separate experiments. Statistical significance of the differences between each treatment and control group (*P<0.05) was determined by unpaired two-sample Student's *t*-test. (**f**) Immunoblottings for p-Smad2/3. The cells were transfected with control siRNA or FPN siRNA and continuously exposed to LY294002 followed by TGFβ1 treatment for 20 min. For **b** and **c**, data represent the mean±s.e.m. of three separate experiments. Statistical significance of the differences between each treatment and TGFβ1 group (*P<0.05, **P<0.01) was determined by unpaired two-sample Student's *t*-test. For **d** and **f**, data represent the mean±s.e.m. of three separate experiments. Statistical significance of the differences between each treatment and TGFβ1 group (*P<0.05, **P<0.01) or TGFβ1+hepcidin (^#^*P*<0.05, ^##^*P*<0.01) was determined by ANOVA (Bonferroni's method).

As hepcidin binding degrades FPN in HSCs, we hypothesized that a deficiency in FPN inhibits TGFβ1 as does hepcidin. As expected, short interfering RNA (siRNA) knockdown of FPN attenuated the induction of TGFβ1 gene in LX-2 cells, whereas over-expression of FPN had the opposite effect (Fig. 8a). Consistently, decrease of FPN specifically prevented the ability of TGFβ1 to induce Smad3 phosphorylation (that is, a little effect on Smad2 phosphorylation), whereas FPN over-expression further enhanced it (Fig. 8b). The results were comparable to those obtained with hepcidin (Fig. 5b). Since FPN acts as an iron exporter, the effect of iron content on TGFβ signalling in HSCs was assessed. Hepcidin treatment increased the intracellular ferritin content (an iron storage marker) in murine HSCs (Supplementary Fig. 7), reflecting the ability of hepcidin to regulate intracellular iron. In addition, ferric ammonium citrate (FAC) treatment attenuated TGFβ1-mediated Smad3 phosphorylation (Fig. 8c). Likewise, deferoxamine (an iron chelator) treatment reversed the hepcidin effect (Fig. 8d). These results support the notion that hepcidin (or FPN knockdown) inhibition of TGFβ signalling may depend on a change in cellular iron content. Next, we examined the effect of FPN modulation on Akt activity. FPN knockdown elevated p-Akt level, as did hepcidin (Fig. 8e). Consistently, the enforced expression of FPN, representing the condition of low intracellular iron, decreased it. FAC treatment enhanced p-Akt level (Fig. 8e). Moreover, PI3K inhibition abrogated the ability of FPN siRNA to inhibit Smad3 phosphorylation (Fig. 8f). Taken all together, our results demonstrate that the inhibition of TGFβ1 signalling by hepcidin may result from Akt-mediated suppression of Smad3 phosphorylation, as a consequence of the degradation of FPN over-expressed in activated HSCs.

### Inhibition of liver fibrosis by HSC-specific deletion of FPN

**Figure 9:**
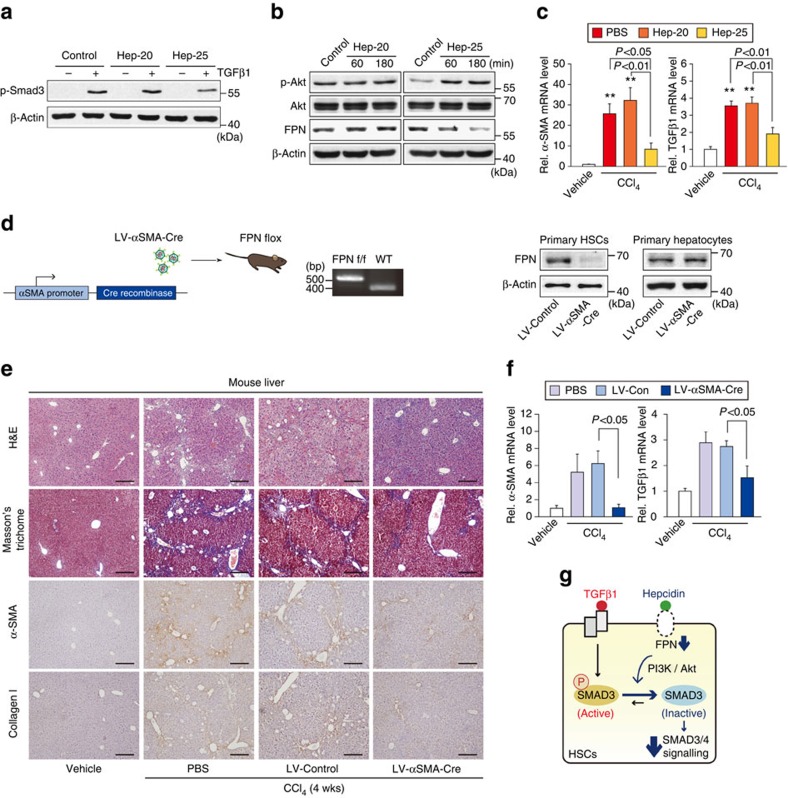
Inhibition of liver fibrosis by HSC-specific deletion of FPN. (**a**) Immunoblottings for p-Smad3. LX-2 cells were treated with 100 nM Hep-20 or Hep-25 for 3 h, and continuously treated with 5 ng ml^−1^ TGFβ1 for 20 min. (**b**) Immunoblottings for p-Akt and FPN in LX-2 cells treated with Hep-20 or Hep-25. (**c**) qRT–PCR assays for α-SMA and TGFβ1. Mice were treated with vehicle or CCl_4_ (0.6 ml kg^−1^ body weight, i.p., 24 h) 3 h after an intraperitoneal injection of 50 μg Hep-20 or Hep-25 (*N*=4 or 6 each). Details for treatment schedule are provided in the Methods section. (**d**) HSC-specific FPN knockout mouse model. FPN-floxed mice were treated with vehicle or CCl_4_ (0.6 ml kg^−1^ body weight, i.p., twice a week, for 4 weeks) after a tail vein injection of PBS, LV-Control or LV-αSMA-Cre (*N*=4 or 6 each). Details for treatment schedule are provided in the Methods section. Immunoblottings for FPN confirmed HSC-specific silencing of FPN. (**e**) H&E, Masson's trichrome stainings and IHC for α-SMA or collagen type 1 on the liver tissues of mice treated as in **d** (scale bar, 100 μm). (**f**) qRT–PCR assays for α-SMA and TGFβ1 in the liver of mice as in **d**. For **c** and **f**, data represent the mean±s.e.m (*N*=4 or 6). Statistical significance of the differences between each treatment group and vehicle (**P<0.01) was determined by analysis of variance (Bonferroni's or LSD method). (**g**) A proposed scheme illustrating the effect of hepcidin on FPN-mediated Smad3 activation in HSCs. Hepcidin inhibits TGFβ1-mediated Smad3 phosphorylation by degrading FPN in HSCs, which relies on Akt signalling. The inhibition of HSC response to TGFβ1 may contribute to anti-fibrotic effect of hepcidin.

To further corroborate the role of FPN in hepcidin effect on liver fibrosis, two additional models were used: truncated hepcidin treatment model and HSC-specific FPN KO mouse models. Since N-terminal five amino acids are involved in the binding of hepcidin to FPN^20^, we used a truncated hepcidin peptide that does not bind to FPN (five N-terminal amino acids-deleted hepcidin, Hep-20), and found that Hep-20 treatment failed to inhibit the Smad3 phosphorylation in LX-2 cells (Fig. 9a). Consistently, Hep-20 had no effect on p-Akt and FPN (Fig. 9b). Similar results were obtained for α-SMA and TGFβ1 in a CCl_4_-treated animal model (Fig. 9c).

Finally, we introduced a genetic model using the FPN-floxed mouse. Lentiviral gene delivery of Cre recombinase (LV-αSMA-Cre), which is activated by the α-SMA promoter, to FPN-floxed mice silenced FPN expression specifically in HSCs, but not in hepatocytes (Fig. 9d). In H&E and Masson's trichrome staining assays, LV-αSMA-Cre injection ameliorated CCl_4_-induced liver fibrosis (Fig. 9e). In addition, α-SMA or collagen-staining intensities were diminished. LV-Control had no effect. qRT–PCR assays for α-SMA or TGFβ1 verified the inhibitory effect of HSC-specific deletion of FPN on fibrogenic gene induction (Fig. 9f). All of these results strengthen the conclusion that hepcidin regulation of FPN (that is, decrease of FPN) in HSCs indeed contributes to the anti-fibrotic effect in the liver (Fig. 9g).

## Discussion

A fundamental question in liver biology is how hepatocytes exert pre-eminent regulation of neighbouring non-parenchymal cells^2^,^4^,^21^. Here we discovered hepcidin as a novel hepatokine to inhibit the HSC activation culminating in liver fibrosis. Investigation of hepcidin expression in the liver of patients with fibrosis or different experimental murine models revealed that the hepcidin expression was downregulated in the fibrotic liver, and inversely correlated with the disease progression; this event was particularly associated with the activation of HSCs. In the present study, we showed for the first time that the adenoviral delivery of hepcidin notably diminished hepatic fibrosis with recovery of liver function in CCl_4_- or BDL-induced animal models of liver fibrosis, indicating that the restoration of hepcidin expression could effectively inhibit the disease progression. In addition, decrease of hepcidin was observed in an animal model with chemical-induced hepatitis, whereas hepcidin over-expression prevented the early fibrogenic event initiated by acute liver injury. Consistently, hepcidin KO mice fed an iron-overload diet displayed chronic liver injury associated with the iron accumulation^22^. All of the outcomes support the hypothesis that hepcidin prohibits the development and progression of liver fibrosis.

Hepcidin expression is likely to be coordinately regulated by complex mechanisms in response to diverse pathophysiological stimuli^8^. In our supplementary analysis, we found that several regulators for hepcidin expression were changed in cirrhosis patients (GEO accession code GSE25097; Supplementary Fig. 8). Of them, CCAAT/enhancer-binding protein-α (C/EBPα), a transcription factor constitutively expressed in hepatocytes, contributed to hepcidin gene induction^23^. Our analysis of GSE25097 database showed the repression of C/EBPα in cirrhosis patients with a reciprocal increase in hepatocyte growth factor that represses hepcidin expression^24^. In addition, the levels of hemojuvelin and transferrin receptor 2, cell surface molecules regulating hepcidin expression^25^, were lower in the cirrhosis patients than in the healthy subjects (Supplementary Fig. 8). Hemojuvelin or transferrin receptor 2 levels inversely correlated with TGFβ1. Thus, it is highly likely that hepatocyte dysfunction and the consequent decreases in C/EBPα and/or cell surface molecules cause hepcidin downregulation in response to pathophysiological stimuli.

HSCs play a major role in the aberrant ECM accumulation after repetitive liver injury, facilitating liver fibrosis. An important finding of our study is identification of the paracrine effect of hepcidin produced by healthy hepatocytes on HSCs. This concept is reinforced by the findings that hepcidin was expressed primarily in hepatocytes, but not in HSCs, and that hepatocyte co-culture prevented HSC activation. This contention was strengthened by antibody neutralization. Among fibrogenic mediators, TGFβ1 is the most potent activator of HSCs^5^,^12^. Recombinant hepcidin treatment lessened TGFβ1-inducible fibrogenic changes in HSCs, as further supported by the result of other cell-based assays; the data of experiments using CM from the culture of hepatocytes indicates that hepcidin originated from healthy hepatocytes inhibits TGFβ1 response in HSCs. However, hepcidin treatment did not significantly alter apoptosis or proliferation of HSCs. Thus, the anti-fibrotic effect of hepcidin may arise from the inhibition of TGFβ1 and its downstream gene expression in HSCs.

Smad2 and Smad3 are the key transcription factors required for the HSC activation and fibrogenic responses^12^. TGFβ1 receptor activation triggers Smad2 phosphorylation in quiescent HSCs, whereas it did so for Smad3 in transdifferentiated HSCs^26^. In addition, Smad2 expression is upregulated by endoplasmic reticulum stress in HSCs^27^. Another important finding of our study is the ability of hepcidin to predominantly inhibit Smad3, as indicated by its phosphorylation, binding of Smad3 and Smad4 and their nuclear translocation. So, the inhibition of HSC activation and TGFβ1 signalling by hepcidin is likely due to its selective effect on Smad3, but not Smad2.

In the current study, we found the novel regulatory effect of hepcidin on Akt signalling in HSCs and the subsequent inhibition of Smad3 phosphorylation. Hepcidin treatment increased p-Akt levels in HSCs, and the inhibition of Akt abrogated the ability of hepcidin to suppress p-Smad3. The selective effect of the Akt (or PI3K) inhibition on the phosphorylation of Smad3, but not Smad2, is in line with the differential effect of hepcidin on the Smads. Apparently, both Akt1 and Akt2 were involved in hepcidin effect on p-Smad3 in HSCs. It is also noteworthy that Akt-mediated Smad3 inhibition by hepcidin was observed in primary hepatocytes (Supplementary Fig. 9a,b), suggesting the possible contribution of autocrine and anti-fibrotic effect of hepcidin.

Another important finding of our study is discovery of the presence of FPN in HSCs in the human liver and the role of FPN in hepcidin inhibition of liver fibrosis. Under the resting state, FPN expression was superior in hepatocytes to HSCs, presumably because hepatocytes are a reservoir of iron storage and mobilization^28^. Of note, FPN was co-localized in α-SMA-positive cells in the liver of fibrosis patients. In addition, FPN expression was raised on CCl_4_ treatment challenge, which was diminished by Ad-Hep delivery. Moreover, either hepatocyte co-culture or exogenous hepcidin treatment lowered FPN expression in HSCs, revealing a close link between the FPN over-expression and HSC activation, as corroborated by the findings that FPN increased TGFβ1 response and that the hepcidin inhibition of TGFβ1 relied on the FPN degradation. The functional effect of FPN in HSCs on hepatic fibrosis was strengthened by the results of animal experiments using a truncated form of hepcidin (Hep-20) and HSC-specific FPN KO mice. Hence, the inhibition of Smad3 by hepcidin resulted from the FPN degradation, which depended on the recovery of p-Akt in the activated HSCs. Our findings demonstrate the novel function of hepcidin in communications between different cell types, providing new information on the antagonism between hepcidin and FPN in HSCs.

Since FPN is an iron exporter, an alteration in intracellular iron content would be a rational basis underlying the inhibition of TGFβ1 signalling. The results shown in this study support the notion that the hepcidin regulation of FPN in HSCs and the resultant change in cellular iron content may lead to the inhibition of TGFβ1 signalling. Our findings that FAC treatment specifically inhibited p-Smad3 with increase of p-Akt, as did hepcidin, and that iron chelator treatment abrogated hepcidin effect on Smad3 support this hypothesis. The proposed concept is in line with the report that cellular iron status affects the Akt activity^29^. It is also possible that excess free iron augments oxidative stress, assisting HSC activation^30^. So, the expected FPN effect on iron exportation in HSCs should be carefully interpreted; iron at the level accumulated in HSCs due to FPN degradation may precisely inhibit the Smad3 pathway in the absence of oxidative burst.

Phosphatase and tensin homologue (PTEN) negatively regulates PI3K and Akt signalling^31^. In a supplementary experiment, we assessed the effect of hepcidin (or FPN modulations) on PTEN. Either hepcidin treatment or FPN siRNA transfection suppressed PTEN, whereas HA-FPN over-expression increased it (Supplementary Fig. 5b). Moreover, FAC treatment also diminished PTEN expression. In our experiment, the receptor-mediated effect of hepcidin on PTEN/Akt was relatively short (<3 h), and the chemical inhibitors of Akt and PI3K were employed to reverse this phenomenon. In other studies, using dominant negative mutant of PI3K or modulations of microRNAs, Akt inhibition was associated with the suppression of HSCs^32^,^33^. The discrepancy may be due to differences in the time course of the Akt inhibition and/or experimental approaches.

Although liver cirrhosis is a global health concern, no drugs have been definitively approved for the disease. Our results demonstrate that hepcidin prohibits liver fibrosis by suppressing TGFβ1–Smad3 pathway in HSCs, which may depend on PI3K/Akt signalling elicited by FPN deficiency. Since hepcidin level is disturbed during the course of liver fibrosis and the gene delivery of hepcidin prevents the disease progression, administration of exogenous hepcidin and/or conditions favoring hepcidin production may be effective in treating fibrosis. We additionally found that hepcidin had no protective effect on hepatocytes (Supplementary Fig. 10), excluding the possibility that hepatoprotective effect influences pathogenesis of liver fibrosis. Liver injury is amplified by inflammatory cytokines, and this event precedes liver fibrosis. It is well recognized that activated HSCs produce TGFβ1 or other cytokines that have cytotoxic effects. Thus, activated HSCs facilitated hepatocyte injury via cytokine production, which reinforces HSC activation^5^. This event makes a positive feed-forward loop for fibrosis. Macrophages have distinct and opposing roles during liver injury and repair, showing macrophage heterogeneity in liver fibrosis; it may involve not only inflammation but also fibrosis resolution^34^. Hepcidin treatment inhibits acute cytokine-induced inflammatory responses in macrophages against endotoxin^35^. On the other hand, activated HSCs induce the differentiation of macrophages to pro-inflammatory and pro-fibrotic phenotype^36^. Thus, the inhibition of HSCs by hepcidin may be additionally of help in inhibiting fibrosis by lowering macrophage activation. Our observation that hepcidin diminished the development of liver fibrosis clearly shows the role of hepcidin in the inhibition of HSC activation during the fibrogenesis.

In a hepcidin-deficient mouse model of hemochromatosis, a synthetic minipeptide of hepcidin has been proposed for the use of preventing iron overload^37^. It has also been claimed that the serum hepcidin:ferritin ratio may be a potential marker for chronic liver disease^38^. Moreover, the established effect of hepcidin on iron homoeostasis is also a merit as anti-fibrotic candidate, since iron overload is frequently observed with hepcidin dysregulation in patients with various liver diseases^39^,^40^,^41^,^42^. Our results shown here support the concept that hepcidin-inducing modulators may be utilized to ameliorate fibrosis and are suggestive of the therapeutic potential of the agents. Finally, our findings, together with the urinary excretion of hepcidin^43^, offer the possibility that hepcidin can serve a diagnostic indicator for liver fibrosis and/or can determine the prognosis of fibrosis disease progression as a non-invasive biomarker.

## Methods

### Materials

Specific antibodies directed against p-Smad2 (3101, 1:2,000 dilution), p-Smad3 (9520, 1:2,000 dilution), Smad2/3 (3102, 1:2,000 dilution), Smad3 (9523, 1:2,000 dilution), p-Akt (9275, 1:2,000 dilution) and Akt (4685, 1:2,000 dilution) were purchased from Cell Signaling (Beverly, MA, USA). Anti-hepcidin and anti-collagen I (ab34710) antibodies were obtained from Abcam (Cambridge, MA, USA; ab30760 for human hepcidin or ab81010 for murine form). Anti-FPN (MTP11-A, 1:2,000 dilution) and anti-α-SMA (ab85370, 1:2,000 dilution) antibodies were supplied by Alpha Diagnostic International (San Antonio, TX, USA) and Abcam (Cambridge, MA, USA), respectively. Anti-β-actin antibody (A5441, 1:10,000 dilution) and other reagents were provided by Sigma-Aldrich (St. Louis, MO, USA). Synthetic human or murine hepcidin recombinant peptide (25 or 20 amino acids) and recombinant TGFβ1 were purchased from Peptides International (Louisville, KY, USA) and R&D Systems (Minneapolis, MN, USA), respectively.

### Patient samples

Human samples with mild (Ishak fibrosis score of three or less, *N*=20) or severe fibrosis (score of four to six, *N*=20) reported in the previous studies were used^27^,^44^. The patients had been diagnosed with liver fibrosis or cirrhosis by histologic examination and ultrasonography in seven different hospitals in South Korea^45^.

### Animal models and experiments

Animal experiments were conducted in accordance with the guidelines of the Institutional Animal Care and Use Committee at Seoul National University. Male C57BL/6 mice or Sprague–Dawley rats at 5 weeks of age were purchased from Charles River Orient (Seoul, Korea) or Samtako Company (Osan, Korea). FPN-floxed mice (129S-Slc40a1tm2Nca/J) were obtained from The Jackson Laboratory (Stock No: 017790). The animals housed at 20±2 °C with 12 h light/dark cycles and a relative humidity of 50±5% under filtered, pathogen-free air, with food and water available *ad libitum*. Six-week-old male C57BL/6 mice were injected with vehicle or CCl_4_ (0.6 ml kg^−1^, intraperitoneally (i.p.)) twice a week for 6 weeks. Six-week-old male Sprague–Dawley rats were i.p. injected with 10 μl kg^−1^ dimethylnitrosamine , three times per week for 4 weeks. To examine the effect of hepcidin on liver fibrosis, we used different mouse fibrosis models: CCl_4_ treatments and BDL. To make a toxicant-induced model, 6-week-old male C57BL/6 mice were i.p. injected with vehicle or 0.6 ml kg^−1^ CCl_4_ twice a week for 6 weeks. At 2 weeks after the first injection, the mice were randomly divided into three groups and were injected with phosphate-buffered saline (PBS), pAd.CMV.GFP (Ad-GFP) or pAd.CMV.Hamp.ires.GFP.Wpre (Ad-Hep) via the tail vein (1 × 10^10^ particle units of each virus in 200  μl PBS per mouse) three times at an interval of 10 days during the remaining period. The mice were killed 2 days after the final CCl_4_ injection. For BDL model, 8-week-old male C57BL/6 mice were infused with Ad-GFP or Ad-Hep via the tail vein 2 days after sham- or BDL operation. The mice were killed 2 weeks after the viral injection. Another set of 8-week-old male C57BL/6 mice were administered with a single dose of CCl_4_ (or vehicle) 6 days after a tail vein injection with Ad-GFP or Ad-Hep, and were killed 24 h afterward. To assess the dose-dependent effect of hepcidin, 8-week-old male C57BL/6 mice were subjected to a single dose of CCl_4_ (or vehicle) 6 days after a tail vein injection of varying doses of Ad-Hep (1 × 10^9^, 3 × 10^9^ or 1 × 10^10^ particle units in 200  μl PBS per mouse) and were killed 24 h afterward.

To specifically delete FPN in HSCs, 6-week-old male FPN-floxed mice were injected with PBS, LV-Control or LV-αSMA-Cre via the tail vein (1 × 10^8^ transducing units of each virus in 200  μl PBS per mouse). Beginning from 1 week after the injection, the mice were i.p. injected with vehicle or 0.6 ml kg^−1^ CCl_4_ twice a week for 4 weeks, and were killed 2 days after the final CCl_4_ injection. Blood and liver samples were taken for serum biochemical and histopathological analyses in all animal experiments. Randomization and blinding strategy was used whenever possible. Animal cohort sizes were determined on the basis of similar previous studies.

### Targeted gene delivery

The LV construct encoding for Cre recombinase was obtained from Addgene (Cambridge, MA, USA). The 3.6-kb regulatory region containing −1.1 kb to ∼+2.5 kb of mouse α-SMA gene was excised from the pSMP8 plasmid^46^, a kind gift from Dr J.A. Fagin (Memorial Sloan Kettering Cancer Center, NY, USA). The original EF1 promoter of pCDH-EF1-MCS plasmid (System Biosciences, Mountain View, CA, USA) was replaced with the α-SMA promoter^27^. The coding region of Cre recombinase was extracted and cloned downstream of the mouse α-SMA gene promoter. The construct was sequenced to evaluate the integrity of insert, and transfected to HEK293T cells to generate viral particles.

### *In vitro* and *in vivo* experiments using a truncated form of hepcidin

The effects of a non-FPN-binding truncated hepcidin peptide (five N-terminal amino acids-truncated hepcidin, Hep-20) and intact hepcidin (Hep-25) were comparatively evaluated in LX-2 cell and animal models. For *in vivo* experiment, 8-week-old male wild-type C57BL/6 mice were treated with a single dose of CCl_4_ (or vehicle) 3 h after an i.p. injection of PBS, Hep-20, or Hep-25 (50 μg per mouse), and were killed 24 h afterward.

### Immunohistochemistry

Liver specimens were fixed in 10% formalin, embedded in paraffin, cut into 4-μm thick sections and were mounted on slides. Tissue sections were immunostained with the antibody directed against hepcidin, collagen I, FPN or α-SMA as in described in the previous study^44^. Briefly, the paraffin-embedded tissue sections were deparaffinized with xylene and rehydrates with alcohols series. After antigen retrieval was performed, the endogenous peroxidase activity was quenched. The sections were pretreated with 10% normal donkey serum for 40 min to block nonspecific antibody binding and were incubated with the antibodies of interest for overnight at 4 °C. The sections were then treated with 2% normal donkey serum for 15 min and incubated with biotin-SP-conjugated affinity pure donkey anti-mouse IgG or anti-rabbit IgG for 2 h. The labelling was done by using 3,3′-diaminobenzidine. After mounting with Permount solution, the sections were examined using light microscope (DMRE, Leica Microsystems, Wetzlar, Germany), and images were acquired with Fluoview-II (Soft Imaging System GmbH, Muenster, Germany) attached on the microscope.

### RNA preparation from formalin-fixed, paraffin-embedded samples

Total RNA was extracted from macro-dissected formalin-fixed, paraffin-embedded (FFPE) samples with the RNeasy FFPE kit (Qiagen, Tokyo, Japan) according to the manufacturer's instructions. Briefly, the sample sections were deparaffinized with xylene, washed with ethanol and dried. Lysis buffer and proteinase K were added to the dried sections. Binding buffer was added to the lysate and transferred to a gDNA Eliminator spin column (Qiagen) to remove genomic DNA. After removing DNA, 100% ethanol was added to the flow-through. The samples were transferred to an RNeasy MinElute column (Qiagen) that binds total RNA. The purified RNA was eluted with 50 μl of RNase-free water.

### RNA isolation and qRT–PCR assays

Total RNA was extracted using Trizol (Invitrogen, Carlsbad, CA, USA) and was reverse-transcribed using oligo-(dT)_16_ primers to obtain complementary DNA. The complementary DNA was amplified by PCR. qRT–PCR was carried out according to the manufacturer's instructions using a StepOne real-time PCR instrument (Thermo Fisher Scientific) and SYBR Premix Ex Taq II kit (Takara Bio, Shiga, Japan). A melting curve of each amplicon was determined to verify its accuracy. The levels of target mRNAs were normalized to those of glyceraldehyde-3-phosphate dehydrogenase or β-actin. The primer sequences are listed in Supplementary Table 1.

### Hydroxyproline content in the liver

Collagen deposition was measured by determination of hydroxyproline in the liver using the hydroxyproline colorimetric assay kit (Biovision, Milpitas, CA, USA). Briefly, liver samples were homogenized in distilled H_2_O and the homogenates were hydrolysed in concentrated HCl by incubation at 120 °C for 3 h. Hydroxyproline contents in the dried hydrolysates were biochemically assessed according to the manufacturer's instructions.

### Immunoblot analysis

Cells were centrifuged at 3,000 *g* for 3 min and allowed to swell after the addition of lysis buffer in the ice for 30 min. The lysates were centrifuged at 10,000 *g* for 10 min to obtain supernatants. Proteins in lysates were resolved by SDS–polyacrylamide gel electrophoresis, and the immobilized proteins were immunoblotted with the antibodies of interest. Bands were visualized using the ECL chemiluminescence system (GE Healthcare, Buckinghamshire, UK). Equal loading of proteins was verified by immunoblotting for β-actin. Original images of immunoblots are shown in Supplementary Fig. 11.

### Cell culture

The LX-2 cell line (an immortalized human HSC line) was kindly supplied by Dr S. L. Friedmann (Icahn School of Medicine at Mount Sinai, NY, USA), whereas the HepG2 cell line was obtained from American Type Culture Collection (Manassas, VA, USA). Primary HSCs or hepatocytes were isolated from rat or mouse liver^47^. Under anaesthesia with Zoletil, livers were perfused with Ca^2+^-free Hank's buffered salt solution (Invitrogen) for 10 min, followed by continuous perfusion with a 0.1% (wt/vol) collagenase (Sigma, Type IV). The whole liver was removed, and minced in the PBS. Cell suspension was filtered through the gauze, and purified with Percoll. The cells were maintained in Dulbecco's modified Eagle's medium containing 10% fetal bovine serum, 50 U ml^−1^ penicillin and 50 μg ml^−1^ streptomycin at 37 °C in a humidified atmosphere with 5% CO_2_. The cells had been tested for mycoplasma contamination by MycoAlert Mycoplasma Detection Kit (Lonza, Alpharetta, GA, USA). The cell line was authenticated by short tandem repeat profiling, but none of them were done in house.

### Transient transfection and siRNA knockdown

The plasmid-encoding HA-FPN (HA-SLC40A1) was obtained from GeneCopoeia (Rockville, MD, USA). Scrambled siRNA (control) and siRNAs directed against human hepcidin, FPN, Akt1 or Akt2 were supplied by Dharmacon (Chicago, IL, USA). Cells were transfected with 100 pmol siRNA using FuGENE HD (Roche, Indianapolis, IN, USA) according to the manufacturer's instructions.

### Flow cytometric analysis

Apoptosis was assessed by the fluorescein isothiocyanate-Annexin V plus propidium iodide (PI) staining method. The cells were collected by trypsinization. After washing with PBS containing 1% fetal bovine serum, the cells were stained with 5 μl fluorescein isothiocyanate-Annexin V and 2 μg ml^−1^ PI. Cell cycle analysis was done using PI^48^. Fluorescence intensity in the cells was assessed using BD FACSCalibur II flow cytometer and the CellQuest software (BD Biosciences, San Jose, CA, USA). In each analysis, 10,000 gated events were recorded.

### Ferritin measurement

The intracellular iron content was assessed by the determination of ferritin level using the ferritin (ferritin light chain (FTL)) mouse ELISA kit (Abcam, Cambridge, MA, USA). Briefly, the lysates of mouse primary HSCs were reacted with anti-ferritin antibodies adsorbed to the surface of polystyrene microtiter wells, and the subsequent procedures were done according to the manufacturer's instructions.

### Data analysis

Statistically significant differences were assessed by unpaired two-sample Student's *t*-test or one-way analysis of variance tests. For each statistically significant effect of treatment, the Bonferroni's or Fisher's least significant difference (LSD) methods were employed for comparisons between multiple group means. Pearson correlation coefficient (*r*-value) was shown in correlation analyses. Data were expressed as the mean±s.e.m. The criterion for statistical significance was set at *P*<0.05 or *P*<0.01. Statistical analyses were performed using IBM SPSS Statistics 23 software.

### Study approval

Studies using human tissues were reviewed and approved by the Institutional Review Board (IRB) at Seoul National University in accordance with the ethical guidelines of the Helsinki Declaration. All participants individually provided written informed consent^45^. All animal experiments were approved by the Institutional Animal Care and Use Committee at Seoul National University, and carried out according to the guidelines of the committee.

### Data availability

The microarray data referenced during the study have been deposited in GEO under accession code GSE25097. All the other data supporting the findings of this study are available within the article and its Supplementary Information files or are available from the corresponding author on reasonable request.

## Additional information

**How to cite this article**: Han, C. Y. *et al*. Hepcidin inhibits Smad3 phosphorylation in hepatic stellate cells by impeding ferroportin-mediated regulation of Akt. *Nat. Commun.* **7**, 13817 doi: 10.1038/ncomms13817 (2016).

**Publisher's note**: Springer Nature remains neutral with regard to jurisdictional claims in published maps and institutional affiliations.

## Supplementary Material

Supplementary Information Supplementary Figures 1-11, Supplementary Table 1
