# Supplementary material for: Hepcidin inhibits Smad3 phosphorylation in hepatic stellate cells by impeding ferroportin-mediated regulation of Akt
Source: Nat Commun. 2016 Dec 22;7:13817. doi: 10.1038/ncomms13817 (PMC5192182; doi:10.1038/ncomms13817)
Supplement: Supplementary Information — Supplementary Figures 1-11, Supplementary Table 1 [file ncomms13817-s1.pdf]

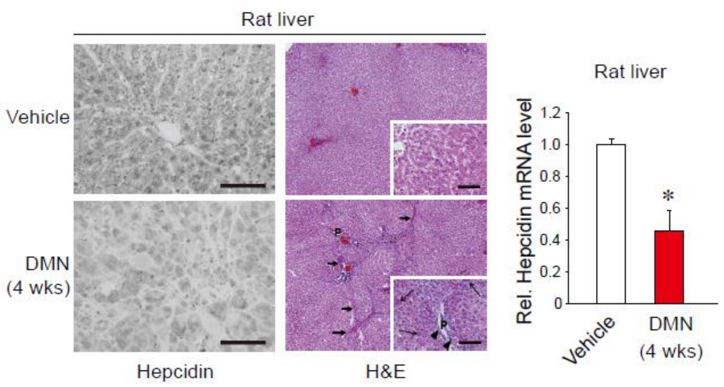

Supplementary Figure 1. Repression of hepcidin expression in the liver of mice treated with DMN

Immunohistochemistry for hepcidin and H&E staining (left). qRT-PCR assays for hepcidin in the liver (right). Rats were treated with vehicle or 10 μl/kg DMN (i.p.) 3 times a week for 4 weeks (N=3, each). Arrows indicate eosinophilic hepatic necrosis, whereas arrowheads do inflammatory cell infiltration in the portal (P) zone (scale bar, 100 μm). Data represent the mean±s.e.m. Statistical significance of the differences between each treatment and vehicle group (*P<0.05) was determined by unpaired two-sample Student's *t*-test.

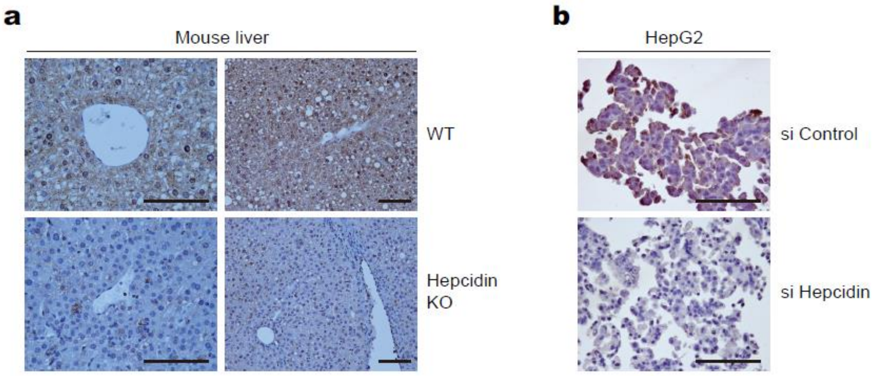

Supplementary Figure 2. Validation of anti-hepcidin antibodies

Immunohistochemistry for hepcidin using anti-mouse hepcidin antibody **(a)** or anti-human hepcidin antibody **(b)** was done on the liver tissue sections from representative wild-type or hepcidin KO mice fed an iron-overload diet for 4 weeks **(a)** or on the pellets of HepG2 cells transfected with control siRNA or hepcidin siRNA for 72 h **(b)**. Scale bar, 100 μm.

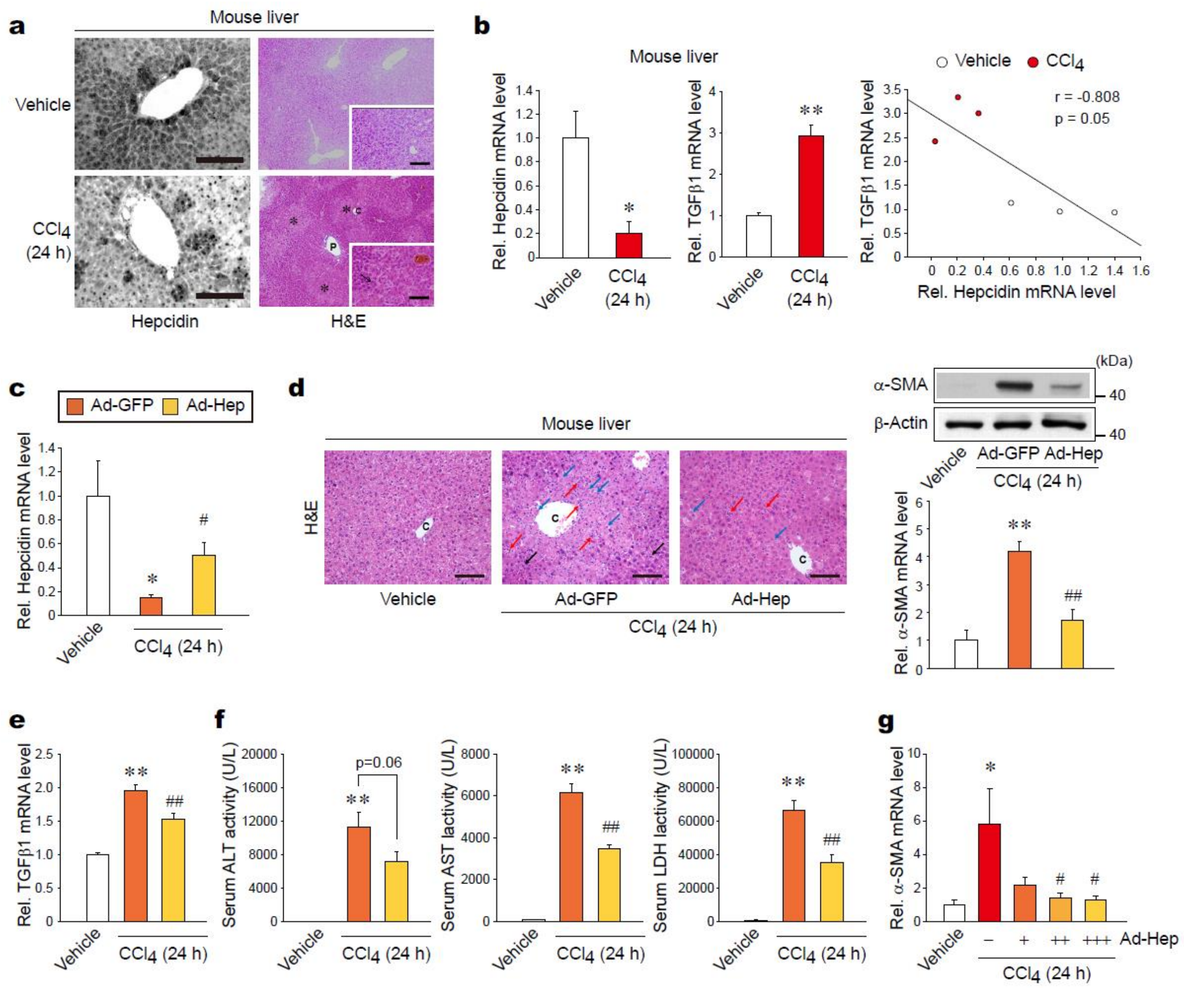

Supplementary Figure 3. Inhibition of the early pathogenic process of fibrosis by hepcidin

(a) Immunohistochemistry for hepcidin and H&E staining. Mice were intraperitoneally injected with a single dose of vehicle or 0.6 ml/kg CCl_4, and the liver samples were obtained 24 h afterward (N=3, each). **(b)** qRT-PCR assays for hepcidin and TGFβ1 (left). A significant inverse correlation existed between hepcidin and TGFβ1 transcript levels (right). Data represent the mean±s.e.m. (N=3 each) Statistical significance of the differences between each treatment and vehicle group (***P*<0.01) was determined by unpaired two-sample Student's *t*-test. **(c)** qRT-PCR assays for hepcidin in the liver. Mice were treated with CCl_4 as in panel **a** at 6 days after tail vein injection of Ad-GFP or Ad-Hep (N=6 or 9 each). **(d)** H&E staining (left). Immunoblottings and qRT-PCR assays for α-SMA in the liver (right). Blue arrows indicate hepatic necrosis, whereas black ones do hepatocyte vacuolization in

intermediate zone. Red arrows represent inflammatory cell infiltration (scale bar, 100 μm). **(e)** qRT-PCR assays for TGFβ1 on the samples obtained as in panel **c**. **(f)** Liver function tests. ALT, AST, and LDH activities were determined on the blood samples. **(g)** qRT-PCR assays for α-SMA in the liver of mice treated as in panel **c** with varying doses of Ad-Hep (N=4 or 6 each). For **c-g**, data represent the mean±s.e.m. Statistical significance of the differences between each treatment group and vehicle (*$P<0.05$, **$P<0.01$), or CCl_4+Ad-GFP ($^{\#}P<0.05$, $^{\#\#}P<0.01$) was determined by ANOVA (Bonferroni's or LSD method).

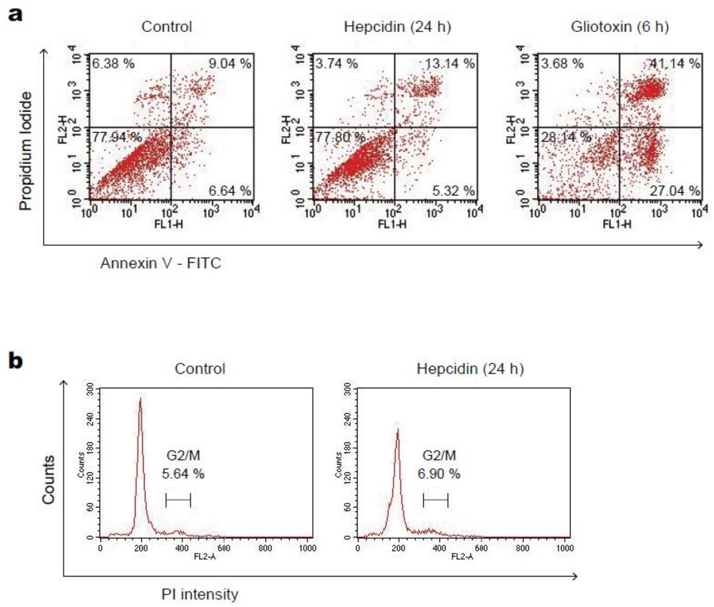

Supplementary Figure 4. Flow cytometry assays for apoptosis or proliferation of HSCs. For apoptosis analysis **(a)**, rat primary HSCs were treated with 100 nM recombinant murine hepcidin for 24 h or 1 μM gliotoxin for 6 h. Gliotoxin was used as a positive control inducing HSC apoptosis. For cell-cycle analysis **(b)**, rat primary HSCs were treated with 100 nM hepcidin for 24 h. Values indicate the percent of G2/M population.

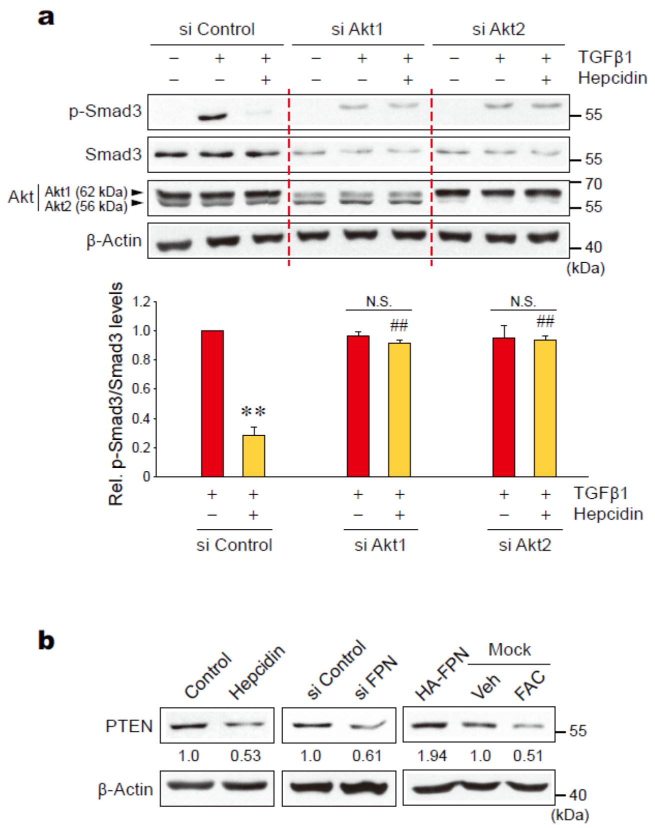

Supplementary Figure 5. Immunoblottings for p-Smad3, Smad3, or PTEN. For **a**, LX-2 cells were transfected with control siRNA, Akt1 or Akt2 siRNA for 48 h, and were exposed to hepcidin for 3 h, followed by TGFβ1 treatment for 20 min. Immunoblottings for Akt confirmed specific knockdown of Akt1 or Akt2. Data represent the mean±s.e.m. of three separate experiments. Statistical significance of the differences between each treatment groups (***P*<0.01) or TGFβ1+hepcidin ($^{\#\#}P$<0.01) was determined by ANOVA (Bonferroni's method) (N.S., not significant). For **b**, LX2 cells were treated with hepcidin for 1 h (left). The cells were also transfected with control siRNA or FPN siRNA for 48 h (middle), or with HA-FPN or Mock for 24 h (right). Mock-transfected cells were treated with vehicle or FAC for 30 min.

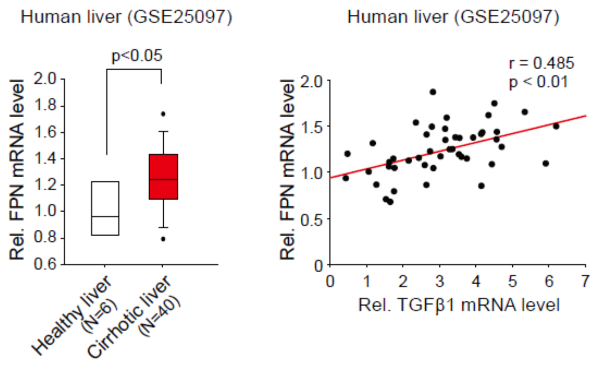

Supplementary Figure 6. FPN mRNA levels in the liver of cirrhosis patients

FPN mRNA levels in the human liver database, GSE25097 (left). A positive correlation between FPN and TGFβ1 transcripts (right). Data were shown as box and whisker plot. Box, interquartile range (IQR); whiskers, 5–95 percentiles; horizontal line within box, median. Statistical significance of the differences between healthy individuals and cirrhosis patients was determined by unpaired two-sample Student's *t*-test.

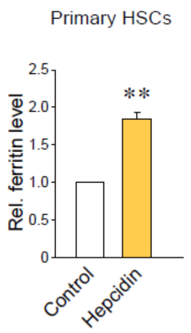

Supplementary Figure 7. The effect of hepcidin on ferritin contents in HSCs

Intracellular ferritin levels were measured in mouse primary HSCs treated with vehicle or 100 nM hepcidin for 24 h. Data represent the mean±s.e.m. of three separate experiments. Statistical significance of the difference between hepcidin treatment and control group (***P*<0.01) was determined by unpaired two-sample Student's *t*-test.

Known Hepcidin regulators	Function	Changes in cirrhosis patients (GSE25097)
TFR2<br>HFE2	Cell surface / Adapter proteins	↓<br>↓
C/EBPα<br>C/EBPβ<br>SMAD4<br>STAT3	Transcription factors	↓<br>–<br>–<br>–
HGF<br>EGF<br>BMP6<br>IL6	Mediators	↑<br>–<br>–<br>–

Human liver (GSE25097)

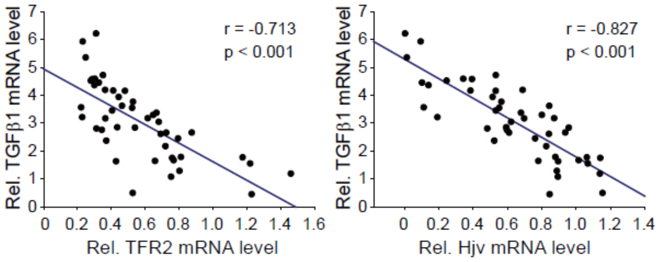

Supplementary Figure 8. Alterations in hepcidin gene regulators in cirrhosis patients

The mRNA levels of known hepcidin gene regulators were comparatively analyzed in the livers of cirrhosis patients and healthy individuals using the GEO database (GSE25097) available in public domain (upper). The arrows represent significant up- or down-regulation (***P*<0.01): statistical significance of the differences between healthy individuals and cirrhosis patients was determined by unpaired two-sample Student's *t*-test. An inverse correlation between TFR2 (or Hjv) and TGFβ1 mRNA (lower).

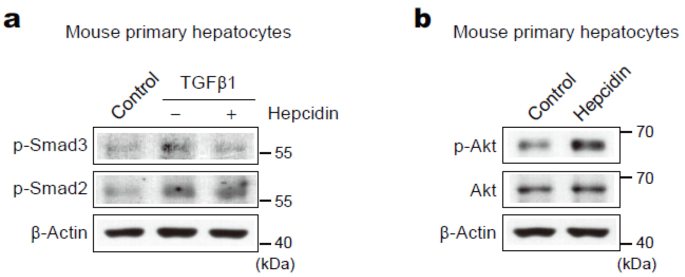

Supplementary Figure 9. Immunoblottings for p-Smad3, p-Smad2, or p-Akt. For **a**, mouse primary hepatocytes were exposed to 100 nM recombinant murine hepcidin for 3 h, and continuously treated with 5 ng/ml TGFβ1 for 20 min. For **b**, the cells were treated with vehicle or 100 nM hepcidin for 1 h.

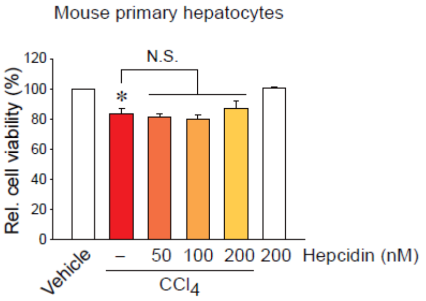

Supplementary Figure 10. Hepcidin effect on the viability of hepatocytes treated with CCl_4

MTT assays were performed in mouse primary hepatocytes exposed to vehicle or 2 mM CCl_4 for 24 h after treatment with varying concentrations of hepcidin for 3 h. Methylene blue (MB 1 μM, for 1 h) was used as a positive control. Data represent the mean±s.e.m. of three separate experiments. Statistical significance of the differences between each treatment and control group (*P<0.05, **P<0.01) or CCl_4 ($^{##}P$<0.01) was determined by ANOVA (Bonferroni's method) (N.S., not significant).

Supplementary Figure 11. Original images of immunoblots

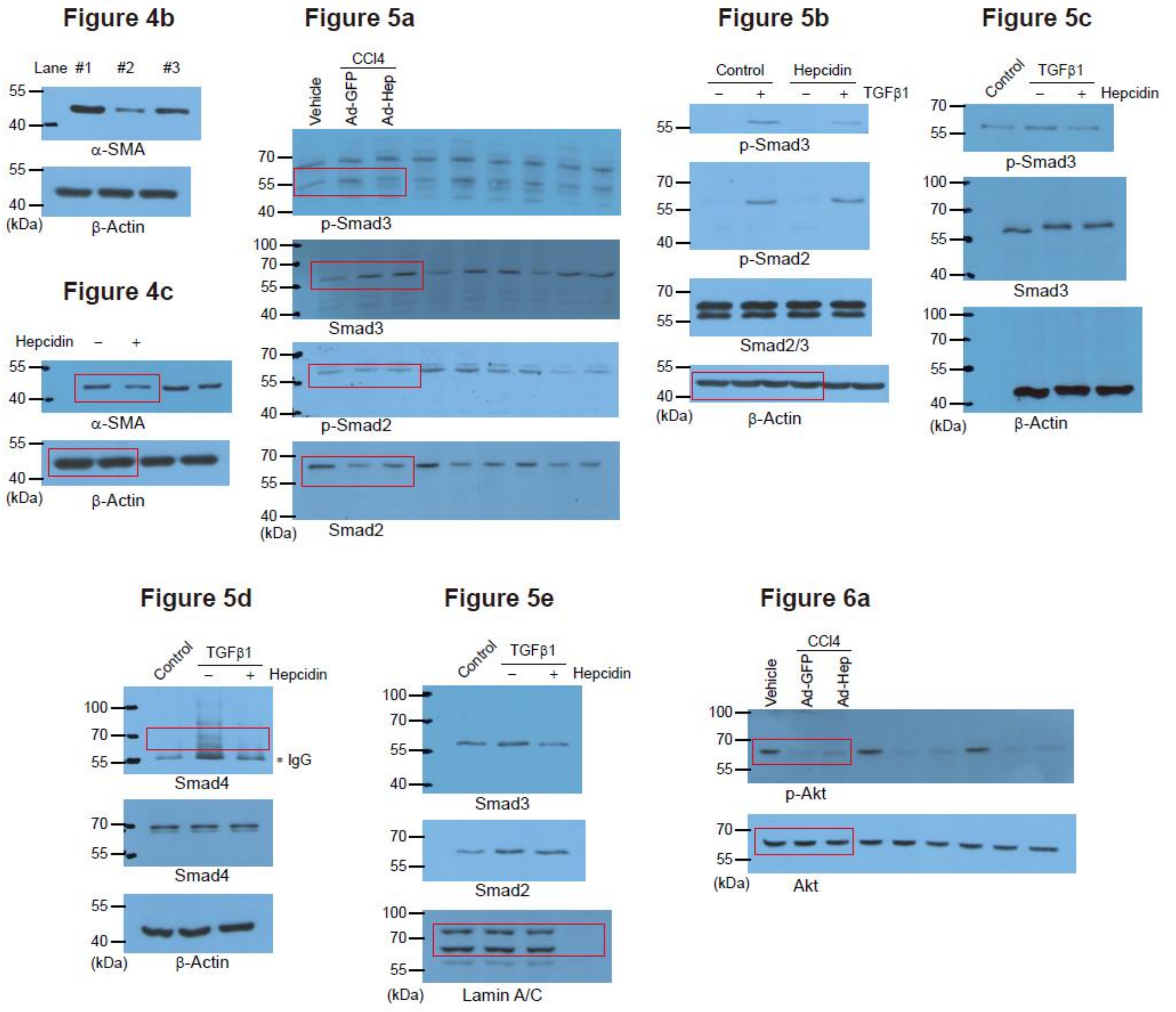

Figure 6b

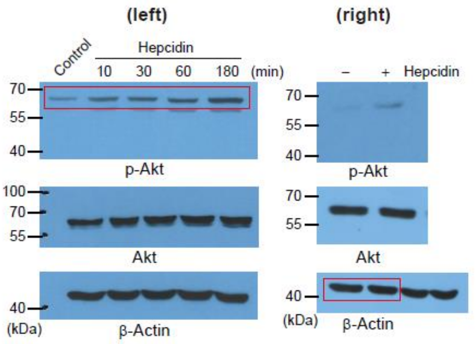

Figure 6c

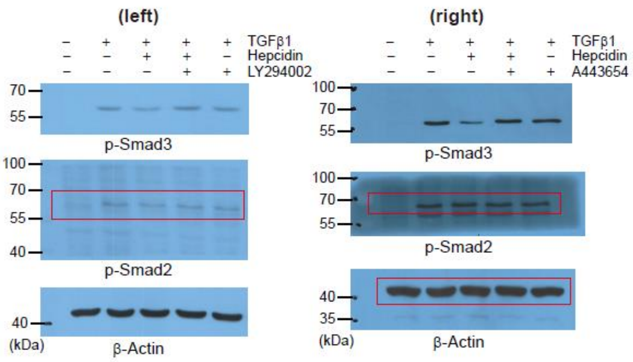

Figure 7b

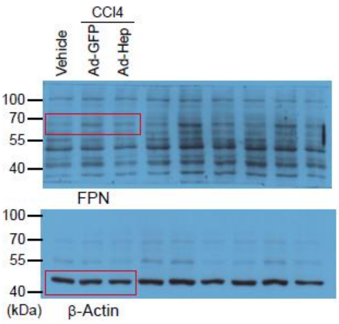

Figure 7c

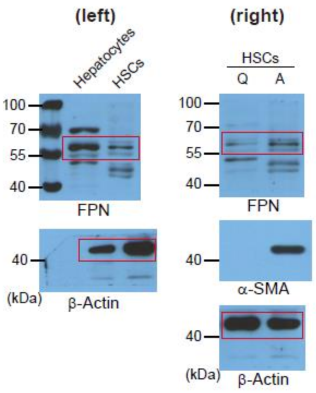

Figure 7d

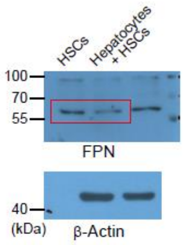

Figure 7e

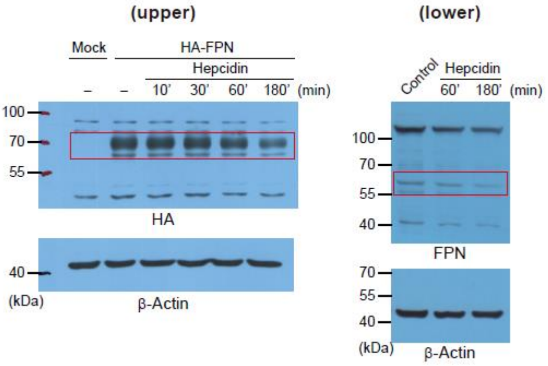

Figure 8b

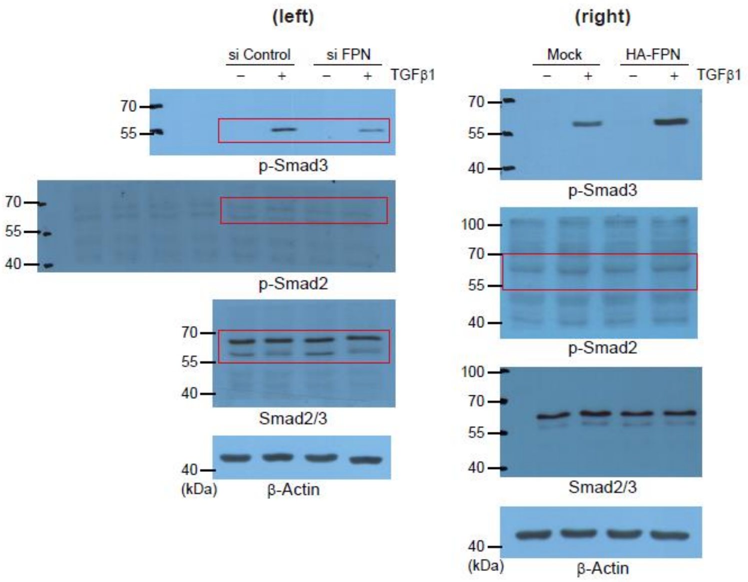

Figure 8c

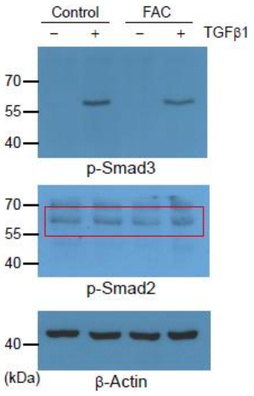

Figure 8d

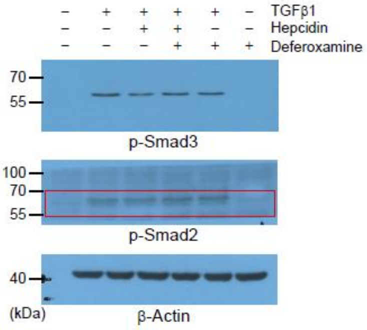

Figure 8e

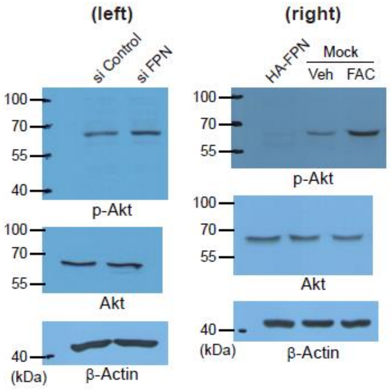

Figure 8f

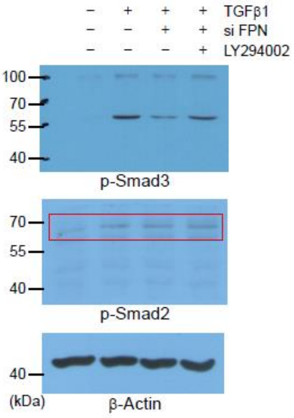

Figure 9a

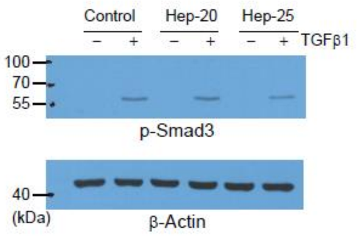

Figure 9b

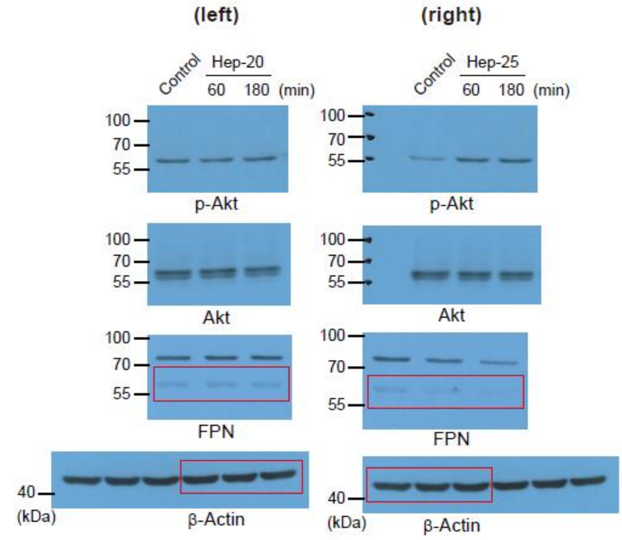

Figure 9d

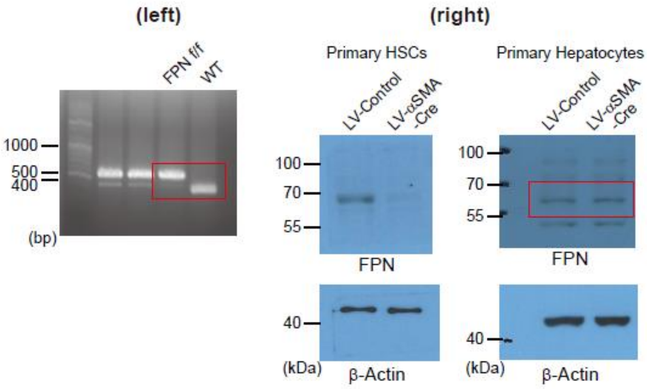

Supplementary Figure 3d

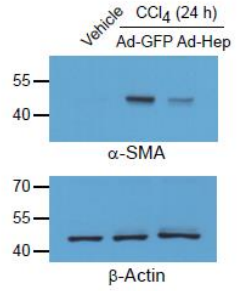

Supplementary Figure 5a

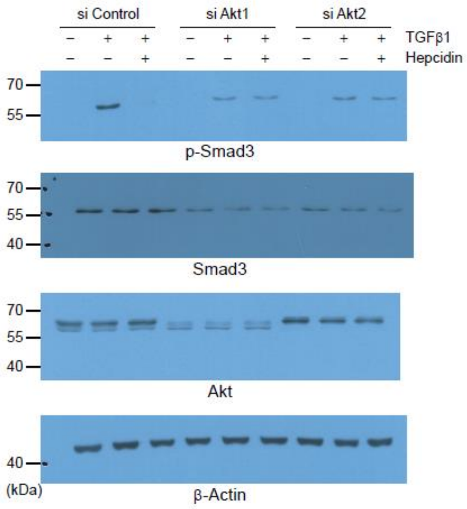

Supplementary Figure 5b

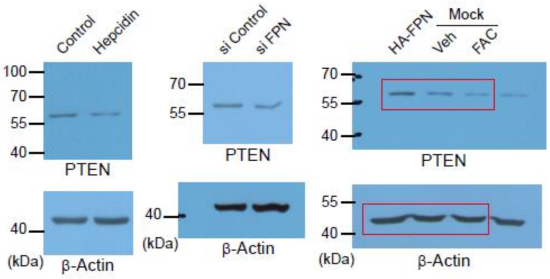

Supplementary Figure 9a

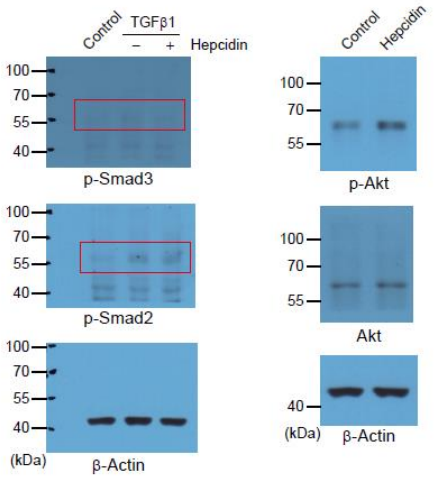

Supplementary Table 1. Primers used in qRT-PCR assays

Species	Genes	Forward (5'-3')	Reverse (5'-3')
Human	Hepcidin (HAMP)	AGCTGGATGCCCATGTTC	CAGCACATCCCACACTTTGA
	TGFβ1	GGCAGTGGTTGAGCCGTGGA	TGTTGGACAGCTGCTCCACCT
	MMP2	GTATTTGATGGCATCGCTCA	CATTCCCTGCAAAGAACACA
	MMP9	CACTGTCCACCCCTCAGAGC	GCCACTTGTCGGCGATAAGG
	COL-1A1	AACATGACCAAAAACCAAAAGTG	CATTGTTTCCTGTGTCTTCTGG
	Ferroportin (SLC40A1)	CAGTTAACCAACATCTTAGC	AAGCTCATGGATGTTAGAG
	GAPDH	GAAGATGGTGATGGGATTTC	GAAGGTGAAGGTCGGAGTC
	β-actin	CTCTTCCAGCCTTCCTTCCTG	CAGCACTGTGTTGGCGTACAG
Mouse	Hepcidin (Hamp1)	CTCCTGCTTCTCCTCCTTGC	GCAATGTCTGCCCTGCTTTC
	α-SMA	GGCTCTGGGCTCTGTAAGG	CTCTTGCTCTGGGCTTCATC
	TGFβ1	GCCCTGGATACCAACTATTGC	GCAGGAGCGCACAATCATGTT
	GAPDH	AACGACCCCTTCATTGAC	TCCACGACATACTCAGCAC
	β-actin	CTGAGAGGGAAATCGTGCGT	TGTTGGCATAGAGGTCTTTACGG
Rat	Hepcidin (Hamp1)	TTCCCCATATGCCTCTTCTG	GGCAGTGTGTTGAGAGGTCA
	PAI-1	TGGTGAACGCCCTCTATTTC	GAGGGGCACATCTTTTTCAA
	Ferroportin (SLC40A1)	TGGATGGGTCCTTACTGTCTGCTAC	TGCTAATCTGCTCCTGTTTTCTCC
	GAPDH	TCCCTCAAGATTGTCAGCAA	AGATCCACAACGGATACATT
